# Isotopy classes for 3-periodic net embeddings

**DOI:** 10.1107/S2053273320000625

**Published:** 2020-03-05

**Authors:** Stephen C. Power, Igor A. Baburin, Davide M. Proserpio

**Affiliations:** a Department of Mathematics and Statistics, Lancaster University, Bailrigg, Lancaster LA1 1SQ, United Kingdom; b Theoretische Chemie, Technische Universität Dresden, D-01062 Dresden, Germany; c Dipartimento di Chimica, Università degli Studi di Milano, Milano 20133, Italy; d Samara Center for Theoretical Materials Science (SCTMS), Samara State Technical University, Samara 443100, Russian Federation

**Keywords:** periodic nets, embedded nets, coordination polymers, isotopy types, crystallographic frameworks

## Abstract

Entangled embedded periodic nets and crystal frameworks are defined, along with their dimension type, homogeneity type, adjacency depth and periodic isotopy type. Periodic isotopy classifications are obtained for various families of embedded nets with small quotient graphs. The 25 periodic isotopy classes of depth-1 embedded nets with a single-vertex quotient graph are enumerated. Additionally, a classification is given of embeddings of *n*-fold copies of **pcu** with all connected components in a parallel orientation and *n* vertices in a repeat unit, as well as demonstrations of their maximal symmetry periodic isotopes. The methodology of linear graph knots on the flat 3-torus [0,1)^3^ is introduced. These graph knots, with linear edges, are spatial embeddings of the labelled quotient graphs of an embedded net which are associated with its periodicity bases.

## Abstract 2

Entangled embedded periodic nets and crystal frameworks are defined, along with their dimension type, homogeneity type, adjacency depth and periodic isotopy type.

## 1. Introduction   

Entangled and interpenetrating coordination polymers have been investigated intensively by chemists in recent decades. Their classification and analysis in terms of symmetry, geometry and topological connectivity is an ongoing research direction (Batten & Robson, 1998 ▸; Carlucci *et al.*, 2003 ▸, 2014 ▸; Blatov *et al.*, 2004 ▸; Alexandrov *et al.*, 2011 ▸). These investigations also draw on mathematical methodologies concerned with periodic graphs, group actions and classification (Delgado-Friedrichs, 2005 ▸; Koch *et al.*, 2006 ▸; Schulte, 2014 ▸; Bonneau & O’Keeffe, 2015 ▸; Baburin, 2016 ▸). On the other hand, it seems that there have been few investigations to date on the dynamical aspects of entangled periodic structures with regard to deformations avoiding edge collisions, or with regard to excitation modes and flexibility in the presence of additional constraints (Guest *et al.*, 2014 ▸). In what follows we take some first steps in this direction and along the way obtain some systematic classifications of basic families.

A proper linear 3-periodic net 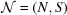 is a periodic bond-node structure in 3D with a set *N* of distinct nodes and a set *S* of noncolliding line segment bonds. The underlying structure graph 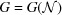 is also known as the topology of 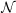 (*cf*. Delgado-Friedrichs *et al.*, 2005 ▸). Thus, the net 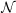 is an embedded net for a topology *G*, it is translationally periodic with respect to each basis vector of some vector space basis for the ambient space, the nodes are distinct points, and the bonds of 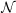 are noncolliding straight-line segments between nodes. We also define the companion structure of a crystallographic bar-joint framework 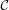. In this case the bonds are of fixed lengths which must be conserved in any continuous motion. Additionally a 3-periodic graph (*G*, *T*) is a pair in which a countable graph *G* carries a specific periodic structure *T*.

Formal definitions of the periodic entities 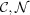 and *G* are given in Definitions 2.1[Statement definition2.1], 2.4[Statement definition2.4] and 2.5[Statement definition2.5]. In crystallographic terminology it is usual in such definitions to require connectedness (Delgado-Friedrichs & O’Keeffe, 2003 ▸). However, we find it convenient in these definitions to extend the usage to cover disconnected periodic structures.

Subclasses of linear *d*-periodic nets 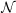 in 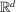 are defined in terms of the diversity of their connected components and we indicate the connections between these class divisions and those used for entangled coordination polymers. In particular, we define the dimension type, which gives a list of the periodic ranks of connected subcomponents, and the homogeneity type which concerns the congruence properties between these components.

Fundamental to the structure of an embedded periodic net are its labelled quotient graphs which are finite edge-labelled graphs determined by periodicity bases. In particular the infinite structure graph 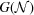 is determined by any labelled quotient graph of this kind, and the (unique) quotient graph 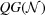 is the graph of the labelled quotient graph of a primitive periodicity basis. These constructs for 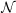 provide useful discriminating features for embedded nets even if they are insensitive to entanglement and catenation.

Our main concern is the entangled nature of linear periodic nets in 3-space which have more than one connected component; however we also consider the self-entanglement of connected structures. Specifically, we approach the classification of linear periodic nets in terms of a formal notion of periodic isotopy equivalence, as given in Definition 6.1[Statement definition6.1]. This asserts that two embedded periodic nets in 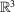 are periodically isotopic if there is a continuous path of noncrossing embedded periodic nets between them which is associated with a continuous path of periodicity bases. In this way we formalize an appropriate variant of the notion of ambient isotopy which is familiar in the theory of knots and links.

As a tool for understanding periodic isotopy we define linear graph knots on the flat 3-torus and their isotopy equivalence classes. Such a graph knot is a spatial graph in the 3-torus which is a geometric realization (embedding) of the labelled quotient graph of a linear periodic net arising from a choice of right-handed periodicity basis for 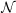. We prove a natural finiteness theorem (Theorem 6.4[Statement theorem6.4]) showing that there are finitely many periodic isotopy types of linear graph knots with a given labelled quotient graph. This in turn implies that there are finitely many periodic isotopy types of linear 3-periodic nets with a given labelled quotient graph.

Our discussions and results are structured as follows. Sections 2 to 6 cover terminology, illustrative examples and general underlying theory. In Section 7 we give group theory methods, while in Sections 8, 9 and 10 we give a range of results, determining periodic isotopy classes and topologies for various families of embedded nets.

More specifically, in Section 2 we give comprehensive terminology, *ab initio*, and give the connections with terms used for coordination polymers and with the net notations of both the Reticular Chemistry Structural Resource (RCSR) (O’Keeffe *et al.*, 2008 ▸) and *ToposPro* (Blatov *et al.*, 2014 ▸). In the key Section 3 we discuss labelled and unlabelled quotient graphs. The example considered in detail in Section 3.1 illustrates terminology and motivates the introduction of model nets for the analysis of periodic isotopy types (periodic isotopes). In Section 4 we define primitive periodicity bases and introduce a measure of adjacency depth for an embedded net. In Section 5, as preparation for the discussion of periodic isotopy for embedded nets, we define linear graph knots on the flat 3-torus 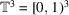 as spatial graphs with (generalized) line segment edges. In Section 6 we discuss various isotopy equivalences for graph knots. Also we define periodic isotopy equivalence for embedded nets and prove that it is an equivalence relation and that there are finitely many periodic isotopes with a common labelled quotient graph. In the group methods of Section 7 we give the group–supergroup construction of entangled nets (Baburin, 2016 ▸), the definition of maximal symmetry periodic isotopes, and the role of Burnside’s lemma in counting periodic isotopes. In Section 8 we determine periodic isotopy classes and also restricted periodic isotopy classes for various multicomponent shift homogeneous embeddings of *n*-fold **pcu**. Such multicomponent embedded nets are related to the interpenetrated structures with translationally equivalent components which are abundant in coordination polymers. For generic embeddings we give proofs based on Burnside’s lemma for counting orbits of spatially equivalent embeddings, while for maximal symmetry embeddings for *n*-**pcu** we use computations based on group–supergroup methods. In Section 9 we give a detailed determination of the 19 topologies and periodic isotopy classes of connected linear 3-periodic nets with a single-vertex quotient graph and adjacency depth 1 (Table 3). In the final section we indicate further research directions.

## 2. Terminology   

In any investigation with cross-disciplinary intentions, in our case between chemistry (reticular chemistry and coordination polymers) and mathematics (isotopy types and periodic frameworks), it is important to be clear of the meaning of terms. Accordingly we begin by defining all terminology from scratch.

The structure graph *G* = (*V*, *E*) of a finite or countably infinite bar-joint framework 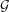 is given *a priori* since, formally, a bar-joint framework 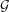 in 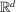 is a pair (*G*, *p*) consisting of a simple graph *G*, the structure graph, together with a placement map 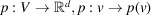. The joints of 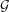 are the points *p*(*v*) and the bars of 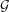 are the (unordered) joint pairs *p*(*v*)*p*(*w*) associated with the edges *vw* in *E*. It is often assumed that *p*(*v*) ≠ *p*(*w*) for the edges *vw* and hence the bars may also be considered to be the associated nondegenerate line segments [*p*(*v*), *p*(*w*)].

A *d*-periodic bar-joint framework in 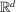 is a bar-joint framework 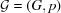 in 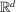 whose periodicity is determined by two sets *F*_*v*_, *F*_*e*_ of noncoincident joints *p*(*v*) and bars *p*(*u*)*p*(*w*), respectively, together with a set of basis vectors for translational periodicity, say 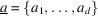. The requirement is that the associated translates of the set *F*_*v*_ and the set *F*_*e*_ are, respectively, disjoint subsets of the set of joints and the set of bars whose unions are the sets of all joints and bars. In particular *p* is an injective map.

The pair of sets (*F*_*v*_, *F*_*e*_) is a building block, or repeating unit, for 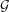. We refer to this pair of sets also as a motif for 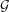 for the basis 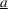 and note that 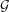 is determined uniquely by any pair of periodicity basis and motif. In fact we shall only be concerned with finite motifs. Also, for 1 ≤ *d*′ < *d* we similarly define a *d*′-periodic bar-joint framework in 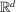 as one that is generated by a finite motif and a linearly independent set of *d*′ vectors for translational periodicity.

**Definition 2.1**

A crystallographic bar-joint framework 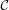 in 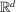, or crystal framework, is a *d*-periodic bar-joint framework in 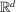 with finitely many translation classes for joints and bars.

Viewing 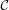 as a bar-joint framework, rather than as a geometric *d*-periodic net, is a conceptual prelude to the consideration of dynamical issues of flexibility and rigidity (Power, 2014*a* ▸), one in which we may bring to bear geometric and combinatorial rigidity theory. Note however that we have not required a crystal framework to be connected.

In the case of a 3D crystal framework 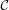, particularly an entangled one of material origin, it is natural to require that the line segments [*p*(*v*), *p*(*w*)], for *vw* ∈ *E*, are essentially disjoint in the sense that they intersect at most at a common endpoint *p*(*x*) for some *x* ∈ *V*. We generally adopt this noncrossing assumption and say that 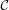 is a proper crystal framework in this case. Thus a proper crystal framework 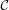 determines a closed set, denoted 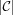, formed by the union of the (nondegenerate) line segments [*p*(*v*), *p*(*w*)], for *vw* ∈ *E*. We call this closed set the body of 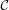. By our assumptions one may recover 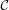 from its body and the positions of the joints.

The connected components of a crystal framework may have a lower rank (or dimension) of periodicity. Accordingly we make the following definition.

**Definition 2.2**

A subperiodic crystal framework, or *d*′-periodic crystal framework, with rank 1 ≤ *d*′ < *d*, is a *d*′-periodic bar-joint framework in 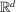, with *d*′ linearly independent period vectors and finitely many translation classes for joints and bars.

For completeness we define a 0-periodic bar-joint framework in 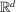 to be a finite bar-joint framework in 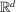. Thus every connected component of a crystal framework in 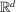 is either itself a crystal framework in 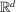 or is a subperiodic crystal framework with rank 1 ≤ *d*′ ≤ *d*, or is a finite framework. Note that a subperiodic subframework exists for 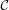 if and only if 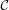 has infinitely many connected components, that is, if and only if the body of 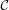 has infinitely many topologically connected components.

In view of the finiteness requirement for the *d*′-periodic translation classes, a subperiodic crystal framework in 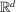 has a joint set consisting of finitely many translates of a sublattice of rank *d*′. In general connected *d*′-periodic subperiodic frameworks may differ in the nature of their affine span, or spatial dimension, which may take any integral value between *d*′ and *d*. Formally, the spatial dimension is the dimension of the linear span of all the so-called bar vectors *p*(*w*) − *p*(*v*) associated with the bars *p*(*v*)*p*(*w*) of the framework. Once again we define a subperiodic framework to be proper if there are no intersections of edges.

The various definitions above, and also the following definition of dimension type, transpose immediately to the simpler category of linear periodic nets 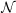, as defined in the next section.

We now introduce the general terminology which is specific to 3D space. Also we indicate how later this formulation of dimension type aligns with the terminology used by chemists for entangled periodic nets.

**Definition 2.3**

A periodic or subperiodic framework 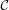 in 3D space has dimension type 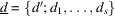, where *d*′ is the periodicity rank of 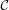 and where *d*_1_, …, *d*_*s*_ is the decreasing list of periodicity ranks of the connected components. (In the symbol, a number representing the periodicity rank of a component is listed only once even if it occurs as the rank of several components.)

In particular there are 15 dimension types *d* for periodicity rank-3 crystallographic frameworks, or for linear 3-periodic nets, namely
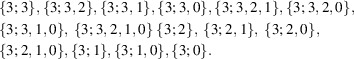



### 2.1. Categories of periodic structures   

Consider the following frequently used terms for periodic structures, arranged with an increasing mathematical flavour: crystal, crystal framework, linear periodic net, periodic graph, topological crystal.

We have defined proper crystal frameworks in 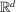 with essentially disjoint bars and these may be viewed as forming an ‘upper category’ of periodic objects for which there is interest in bar-length-preserving dynamics. If we disregard bar lengths, but not geometry, then we are in the companion category of positions, or line drawings, or embeddings of *d*-periodic nets in 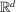. Such embeddings are of interest in reticular chemistry and in this connection we may define a linear *d*-periodic net in 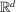 to be a pair (*N*, *S*), consisting of a set *N* of nodes and a set *S* of line segments, where these sets correspond to the joints and bars of a proper *d*-periodic crystal framework. A stand-alone definition is the following.

**Definition 2.4**

A (proper) linear *d*-periodic net in 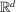 is a pair 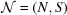 where (i) *S*, the set of edges (or bonds) of 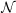, is a countable set of essentially disjoint line segments [*p*, *q*], with *p* ≠ *q*, (ii) *N*, the set of vertices (or nodes) of 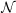, is the set of endpoints of members of *S*, (iii) there is a basis of vectors for 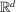 such that the sets *N* and *S* are invariant under the translation group 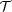 for this basis, (iv) the sets *N* and *S* partition into finitely many 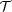-orbits.

Thus a linear periodic net can be thought of as a proper linear embedding of the structure graph of a crystal framework, the relevant crystal frameworks being those with no isolated joints of degree 0. Note that a linear periodic net is not required to be connected.

A linear *d*-periodic net is referred to in reticular chemistry as an embedding of a ‘*d*-periodic net’. This is because the term *d*-periodic net has been appropriated for the underlying structure graph of a linear periodic net. See Delgado-Friedrichs & O’Keeffe (2003 ▸), for example. This reference, to a more fundamental category on which to build, so to speak, then allows one to talk of a *d*-periodic net having an embedding with, perhaps, certain symmetry attributes. It follows then, tautologically, that a *d*-periodic net is a graph with certain periodicity properties and we formally specify this in Definition 2.5[Statement definition2.5].

The next definition is a slight variant of the definition given by Delgado-Friedrichs (2005 ▸), in that we also require edge orbits to be finite in number.

**Definition 2.5**

(i) A periodic graph is a pair (*G*, *T*), where *G* is a countably infinite simple (abstract) graph and *T* is a free abelian subgroup of Aut(*G*) which acts on *G* freely and is such that the sets of vertex orbits and edge orbits are finite. The group *T* is called a translation group for *G* and its rank is called the dimension of (*G*, *T*). (ii) A *d*-periodic graph or a *d*-periodic net is a periodic graph of dimension *d*. (iii) The translation group *T* and the periodic graph (*G*, *T*) are maximal if no periodic structure (*G*, *T*′) exists with *T*′ a proper supergroup of *T*.

The subgroup *T* [or the pair (*G*, *T*)] is referred to as a periodic structure on *G*. Some care is necessary with assertions such as ‘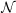 is an embedding of a 3-periodic net *G*’. This has two interpretations according to whether *G* comes with a given periodic structure *T* which is to be represented faithfully in the embedding as a translation group or, on the other hand, whether the embedding respects some periodic structure *T*′ in 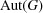.

Finally we remark that there is another category of nets which is relevant to more mathematical considerations of entanglement, namely string-node nets in the sense of Power & Schulze (2018 ▸). In the discrete case these have a similar definition to linear periodic nets but the edges may be continuous paths rather than line segments.

### 2.2. Maximal symmetry, the RCSR and self-entanglement   

Let 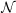 be a linear *d*-periodic net. Then there is a natural injective inclusion map
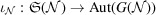
from the usual symmetry group (space group) 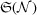 of 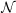 to the automorphism group of its structure graph 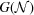.

**Definition 2.6**

Let 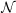 be a linear *d*-periodic net. (i) The graphical symmetry group of 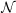 is the automorphism group 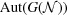. This is also called the maximal symmetry group of 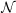. (ii) A maximal symmetry embedding of 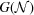 is an embedded net 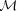 for which 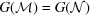 and the map 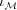 is a group isomorphism.

A key result of the work of Delgado-Friedrichs (2003 ▸, 2005 ▸) shows that many connected 3-periodic graphs have unique maximal symmetry placements, possibly with edge crossings. These placements arise for a so-called stable net by means of a minimum-energy placement, associated with a fixed lattice of orbits of a single node, followed by a renormalization by the point group of the structure graph. In fact a stable net is defined as one for which the minimum-energy placement has no node collisions. See also Delgado-Friedrichs & O’Keeffe (2003 ▸), Sunada (2013 ▸). While maximum-symmetry positions for connected stable nets are unique, up to spatial congruence and rescaling, edge crossings may occur for simply defined nets because, roughly speaking, the local edge density is too high. It becomes an interesting issue then to define and determine the finitely many classes of maximum-symmetry proper placements and this is true also for multicomponent nets. See Section 7.1.

The RCSR (O’Keeffe *et al.*, 2008 ▸) is a convenient online database which, in part, defines a set of around 3000 topologies *G* together with an indication of their maximal symmetry embedded nets. The graphs *G* are denoted in bold notation, such as **pcu** and **dia**, in what is now standard nomenclature. We shall make use of this and denote the maximal symmetry embedding of a connected topology **abc** as 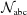. This determines 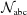 as a subset of 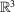 up to a scaling factor and spatial congruence. *ToposPro* (Blatov *et al.*, 2014 ▸) is a more sophisticated program package, suitable for multipurpose crystallochemical analysis and has a more extensive periodic net database. In particular it provides labelled quotient graphs for 3-periodic nets.

Both these databases give coordination density data which can be useful for discriminating the structure graphs of embedded nets.

In Section 6 we formalize the periodic isotopy equivalence of pairs of embedded nets. One of our motivations is to identify and classify connected embedded nets which are not periodically isotopic to their maximal symmetry embedded net. We refer to such an embedded net as a self-entangled embedded net.

### 2.3. Derived periodic nets   

We remark that the geometry and structural properties of a linear periodic net or framework can often be analysed in terms of derived nets or frameworks. These associated structures can arise through a number of operations and we now indicate some of these.

(i) The periodic substitution of a (usually connected) finite unit with a new finite unit (possibly even a single node) while maintaining incidence properties. This move is common in reticular chemistry for the creation of ‘underlying nets’ (Alexandrov *et al.*, 2011 ▸; O’Keeffe & Yaghi, 2012 ▸).

(ii) A more sophisticated operation which has been used for the classification of coordination polymers replaces each minimal ring of edges by a node (barycentrically placed) and adds an edge between a pair of such nodes if their minimal rings are entangled. In this way one arrives at the Hopf ring net (HRN) of an embedded net 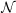 (Alexandrov *et al.*, 2012 ▸, 2017 ▸). This is usually well defined as a (possibly improper) linear 3-periodic net and it has proven to be an effective discriminator in the taxonomic analysis of crystals and coordination polymer databases.

(iii) There are various conventions in which notational augmentation is used (O’Keeffe *et al.*, 2008 ▸) to indicate the derivation of an embedded net or its relationship with a parent net. In the RCSR listing, for example, the notation **pcu-c4** indicates the topology made up of four disjoint copies of **pcu** (O’Keeffe *et al.*, 2008 ▸). In Tables 1 ▸, 2 ▸ we use a notation for model embedded nets, such as 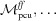, which is indicative of a hierarchical construction.

(iv) On the mathematical side, in the rigidity theory of periodic bar-joint frameworks 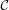 there are natural periodic graph operations and associated geometric moves, such as periodic edge contractions, which lead to inductive schemes in proofs. In particular periodic Henneberg moves, which conserve the average degree count, feature in the rigidity and flexibility theory of such frameworks (Nixon & Ross, 2015 ▸).

### 2.4. Types of entanglement and homogeneity type   

Let us return to descriptive aspects of disconnected linear 3-periodic nets 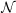 in 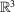.

We first note the following scheme of Carlucci *et al.* (2014 ▸) which has been used in the classification of observed entangled coordinated polymers. Such a coordination polymer, 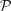 say, is also a proper linear *d*-periodic net 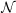 in 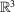, and this is either of full rank *d* = 3, or is of subperiodicity rank 1 ≤ *d* < 3, or is a finite net (which we shall say has rank 0). Let 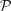 be a *d*-periodic coordination polymer in 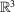. Then 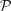 is said to be (i) in the interpenetration class if all connected components of 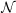 are also *d*-periodic, (ii) in the polycatenation class otherwise.

Thus 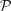 is in the interpenetration class if and only if the dimension type of its net is {3; 3}, {2; 2}, {1; 1} or {0; 0}.

The entangled coordination polymers in the interpenetration class may be further divided as subclasses of *n*-fold type, according to the number *n* of components, where, necessarily, *n* is finite.

The linear 3-periodic nets in the polycatenation class have some components which are subperiodic and in particular they have countably many components. When all the components are 2-periodic, that is, when 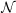 has dimension type {3; 2}, then 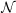 is either of parallel type or inclined type. Parallel type is characterized by the common coplanarity of the periodicity vectors of the components, whereas 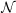 is of inclined type if there exist two components which are not parallel in this manner. The diversity here may be neatly quantified by the number, ν_2_ say, of planes through the origin that are determined by the (pairs of) periodicity vectors of the components.

Similarly, the disconnected linear 3-periodic nets of dimension type {3; 1} can be viewed as being of parallel type, with all the 1-periodic components going in the same direction, or, if not, as inclined type. In fact there is a natural further division of the nonparallel (inclined) types for the nets of dimension type {3; 1} according to whether the periodicity vectors for the components are coplanar or not. We could describe such nets as being of coplanar inclined type and triple inclined type, respectively. The diversity here may also be neatly quantified by the number, ν_1_ say, of lines through the origin that are determined by the periodicity vectors of the components.

The disconnected nets of parallel type are of particular interest for their mathematical and observed entanglement features, such as Borromean entanglement and woven or braided structures (Carlucci *et al.*, 2014 ▸; Liu *et al.*, 2018 ▸).

The foregoing terminology is concerned with the periodic and subperiodic nature of the components of a net without regard to further comparisons between them. On the other hand, the following terms identify subclasses according to the possible congruence between the connected components.


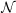
 is of homogeneous type if its components are pairwise congruent. Here the implementing congruences are not assumed to belong to the space group.


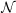
 is *n*-heterogeneous, with *n* > 1, if there are exactly *n* congruence classes of connected components.

Thus every 3-periodic linear net 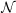 in 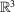 is either of homogeneous type or *n*-heterogeneous for some *n* = 2, 3, ….

The homogeneous linear 3-periodic nets split into two natural subclasses.


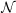
 is of shift-homogeneous type if all components are pairwise translationally equivalent (shift equivalent). Otherwise, when 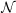 contains at least one pair of components which are not shift equivalent then we say that the homogeneous net 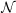 is of rotation type.

Finally we take account of the space group of 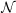 to specify a very strong form of homogeneity: each of the two homogeneous types contains a further subtype according to whether 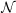 is also of transitive type or not, where 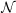 is component transitive (or is of transitive type) if the space group of 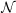 acts transitively on components.

Such component transitive periodic nets have been considered in detail by Baburin (2016 ▸) with regard to their construction through group–supergroup methods.

**Figure 1:**
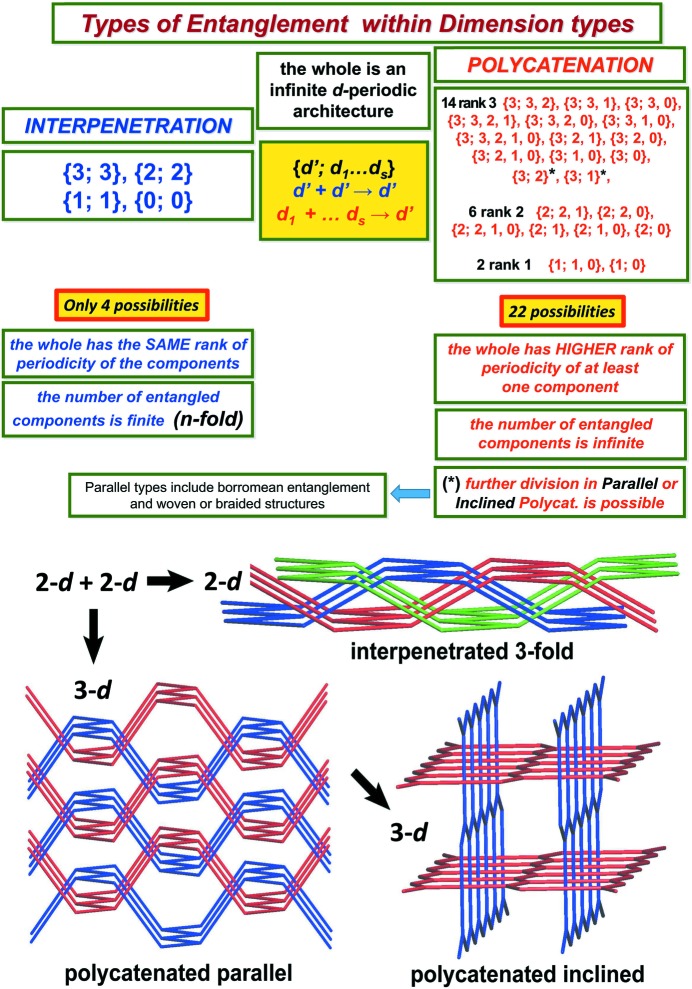
Dimension type and polycatenation.

Note that a homogeneous linear 3-periodic net in 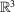 which is not connected falls into exactly one of 16 possible dimension–homogeneity types according to the four possible types of homogeneity and the four possible dimension types 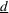, namely 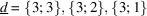 or {3; 0}. For a full list of correspondences see Fig. 1 ▸.

### 2.5. Catenation and Borromean entanglement   

To the dimension–homogeneity type division of multicomponent embedded nets one may consider further subclasses which are associated with entanglement features between the components. Indeed, our main consideration in what follows is a formalization of such entanglement in terms of linear graph knots. We note here some natural entanglement invariants of Borromean type. In fact the embedded nets of dimension type {3; 2} have been rather thoroughly identified by Carlucci *et al.* (2014 ▸), Alexandrov *et al.* (2017 ▸) where it is shown that subdimensional 2-periodic components can be catenated or woven in diverse ways.

To partly quantify this one may define the following entanglement indices. Let 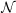 be such a parallel type embedded net, with dimension type {3; 2}, and let 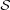 be a finite set of components. Then a separating isotopy of 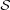 is a continuous deformation of 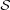 to a position which properly lies on both sides of the complement of a plane in 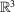. If there is no separating isotopy for a pair 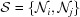 of components of 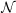 then we say that they are entangled components, or are properly entangled. This partial definition can be made rigorous by means of a formal definition of periodic isotopy. We may then define the component entanglement degree of a component 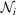 of 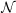 to be the maximum number, 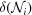 say, of components 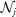 which can form an entangled pair with 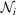. Also the component entanglement degree of 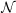 itself may be defined to be the maximum such value.

Likewise, one can define the entanglement degrees of components for embedded nets of dimension type {3; 1} and for the subdimensional nets of dimension type {2; 2} (woven layers) and dimension type {1; 1} (braids). More formally, we may say that 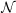 has Borromean entanglement if there is a set of *n* ≥ 3 connected components which admit no separating periodic isotopy while, on the other hand, every pair in this set admits a separating periodic isotopy. In a similar way one can formalize the notion of Brunnian catenation (Liang & Mislow, 1994 ▸) for a multicomponent embedded net.

### 2.6. When topologies are different   

Two standard graph isomorphism invariants used by crystallographers are the point symbol and the coordination sequence.

In a vertex transitive countable graph *G* the point symbol (PS), which appears as 4^24^6^4^ for **bcu** for example, indicates the multiplicities (24 and 4) of the cycle lengths (4 and 6) for a set of minimal cycles which contain a pair of edges incident to a given vertex. If the valency (or coordination) of *G* is *r* then there are *r*(*r* − 1)/2 such pairs and so the multiplicity indices sum to *r*(*r* − 1)/2. For *G* nontransitive on vertices the point symbol is a list of individual point symbols for the vertex classes (Blatov *et al.*, 2010 ▸).

The coordination sequence (CS) of a vertex transitive countable graph *G* is usually given partially as a finite list of integers associated with a vertex *v*, say *n*_1_, *n*_2_, *n*_3_, *n*_4_, *n*_5_, where *n*_*k*_ is the number of vertices *w* ≠ *v* for which there is an edge path from *v* to *w* of length *k* but not of shorter length. For **bcu** this sequence is 8, 26, 56, 98, 152. Cumulative sums of the CS are known as topological densities, and the RCSR, for example, records the tenfold sum, td10.

Even the entire CS is not a complete invariant for the set of underlying graphs of embedded periodic nets. However, this counting invariant can be useful for discriminating nets whose local structures are very similar. A case in point is the pair 8T17 and 8T21 appearing in Table 3 ▸, which have partial coordination sequences 8, 32, 88 and 8, 32, 80, respectively.

## 3. Quotient graphs   

We now define quotient graphs (QGs) and labelled quotient graphs (LQGs) associated with the periodic structure bases of a linear periodic net 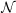. Although QGs and LQGs are not sensitive to entanglement they nevertheless offer a means of subcategorizing linear periodic nets. See, for example, the discussions by Eon (2011 ▸, 2016 ▸), Klee (2004 ▸), Klein (2012 ▸), Thimm (2004 ▸) and Section 4.3 below.

Let 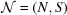 be a linear 3-periodic net with periodic structure basis 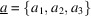. Then 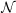 is completely determined by 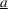 and any associated building block motif (*F*_*v*_, *F*_*e*_). It is natural, especially in illustrating examples, to choose the set *F*_*e*_ of edges of 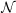 to be as connected as possible and to choose *F*_*v*_ to be a subset of the vertices of these edges. Let 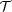 denote the translation group associated with 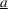, so that 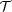 is the set of transformations
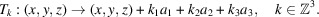
Each (undirected) line segment edge *p*(*e*) in *F*_*e*_ has the form [*T*_*k*_ *p*(*v*_*e*_), *T*_*l*_ *p*(*w*_*e*_)], where *p*(*v*_*e*_) and *p*(*w*_*e*_) are the representatives in *F*_*v*_ for the endpoint nodes *T*_*k*_ *p*(*v*_*e*_), *T*_*l*_ *p*(*w*_*e*_) of the edge *p*(*e*). The labels *k* and *l* here may be viewed as the cell labels or translation labels associated with endpoints of *p*(*e*). [As before *v*_*e*_, *w*_*e*_ indicate vertices of the underlying structure graph 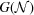.]

The LQG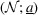 of the pair 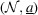 is a finite multigraph together with a directed labelling for each edge, where the labelling is by elements 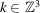. The vertices correspond to (or are labelled by) the vertices *v* of the nodes *p*(*v*) in *F*_*v*_, and the edges correspond to edges *p*(*e*) in *F*_*e*_. The directedness is indicated by the ordered pair (*v*_*e*_, *w*_*e*_), or by *v*_*e*_ *w*_*e*_ (viewed as directedness ‘from *v*_*e*_ to *w*_*e*_’). The label for this directed edge is then *k* − *l* where *k*, *l* are the translation labels as in the previous paragraph, and so the labelled directed edge is denoted (*v*_*e*_ *w*_*e*_, *k* − *l*). There is no ambiguity since the directed labelled edge (*v*_*e*_ *w*_*e*_, *k* − *l*) is considered to be the same directed edge as (*w*_*e*_ *v*_*e*_, *l* − *k*). In particular the following definition of the depth of a labelled directed graph is well defined.

**Definition 3.1**

Let 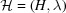 be any LQG. Then the depth of 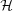 is the maximum modulus of the coordinates of the edge labels.

The QG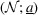 of the pair 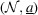 is the undirected graph *G* obtained from the LQG. If 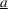 is a primitive periodicity basis, that is, one associated with a maximal lattice in 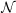, then QG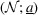 is independent of 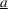 and is the usual quotient graph of 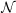 in which the vertices are labelled by the translation group orbits of the nodes. Primitive periodicity bases are discussed further in the next section. Moreover, we identify there the ‘preferred’ primitive periodicity bases which have a ‘best fit’ for 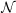 in the sense of minimizing the maximum size of the associated edge labels.

**Definition 3.2**

The QG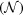 of a linear periodic net 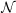 in 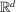 is the unlabelled multigraph graph of the LQG determined by a primitive periodicity basis.

Finally we remark on the homological terminology related to the edge labellings of a LQG. The homology group 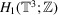 of the 3-torus 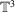 is isomorphic to 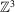. In this isomorphism the standard generators of 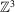 may be viewed as corresponding to (homology classes of) three 1-cycles which wind once around the 3-torus (which we may parametrize naturally by the set [0, 1)^3^) in the positive coordinate directions. Also, we may associate the standard ordered basis for 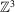 with a periodicity basis 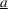 for 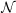. In this case the sum of the labels of a directed cycle of edges in the labelled quotient graph is equal to the homology class of the associated closed path in 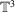.

### 3.1. Embedded nets with a common LQG   

We now consider the family of all linear 3-periodic nets (proper embedded nets) which have a periodic structure basis determining a particular common LQG. This discussion illuminates some of the terminology set out so far and it also gives a prelude to discussions of periodic isotopy. Also it motivates the introduction of model nets and linear graphs knots on the 3-torus.

Let *H* be the 6-coordinated graph with two vertices *v*_1_, *v*_2_, two connecting edges between them and two loop edges on each vertex. (We say that a finite or countable graph is *n*-coordinated if every vertex has valency *n*.) Let (*H*, λ) be the LQG with labels (0, 0, 1), (1, 1, 1) for the loop edges for *v*_1_, labels (0, 1, 0), (0, 0, 1) for the loop edges for *v*_2_, and labels (0, 0, 0), (0, −1, −1) for the two directed edges from *v*_1_ to *v*_2_. Let 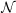 be an embedded net with a general periodic structure basis 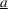 such that LQG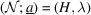. (In particular 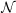 has adjacency depth 1, as defined in the next section.) Note that the four loop edges on *v*_1_ and *v*_2_ imply that 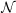 has two countable sets of 2D parallel subnets all of which are pairwise disjoint. These subnets are either parallel to the pair {*a*_2_, *a*_3_} or to the pair {*a*_3_, *a*_1_ + *a*_2_ + *a*_3_}. In particular if 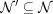 is the embedded net which is the union of these 2D subnets then 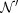 is a derived net of 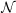 of dimension type {3; 2}. Also 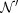 is in the polycatenation class of inclined type (rather than parallel type). By means of a simple oriented affine equivalence (see Definition 4.3[Statement definition4.3]) the general pair 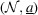 with LQG(*H*, λ) is equivalent to a pair 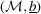, having the same LQG, where 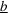 is the standard right-handed orthonormal basis. We shall call the pair 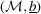 a model net.

**Figure 2:**
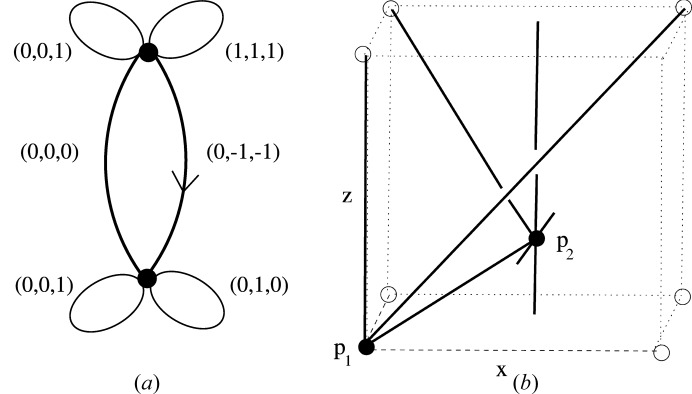
(*a*) A labelled quotient graph (*H*, λ). (*b*) Part of the net 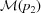 in the cube [0, 1)^3^ where 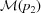 is determined by the LQG together with the standard basis periodic structure 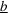, a node *p*_1_ at the origin and the node *p*_2_ in the unit cell [0, 1)^3^.

By translation (another oriented affine transformation) we may assume that there is a node *p*_1_ of 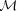 at the origin which is associated with the vertex *v*_1_ of *H*. Let *p*_2_ be the unique node associated with *v*_2_ which lies in the unit cell [0, 1)^3^. Now the pair 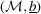 is uniquely determined by *p*_2_ and we denote it simply as 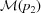. Fig. 2 ▸ illustrates the part of the linear periodic net 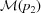 which is visible in [0, 1)^3^. In Section 5 we shall formalize diagrams such as Fig. 2 ▸ in terms of linear graph knots on the flat 3-torus.

With this normalization the point *p*_2_ can be any point in [0, 1)^3^, subject to the essential disjointness of edges, and we write 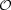 for this set of positions of *p*_2_. Note that as *p*_2_ moves on a small closed circular path around the main diagonal its incident edges are determined and there will be five edge crossings with the diagonal. In fact the two vertical edges and the two horizontal edges which are incident to *p*_2_ contribute two crossings each, and the other edge incident to *p*_2_ contributes one crossing. These are the only edge crossings that occur as *p*_2_ ‘carries’ its six edges of incidence during this motion. It follows from similar observations that 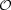 is the disjoint union of five pathwise connected sets.

In this way we see that a pair of nets 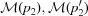, with *p*_2_, *p*_2_′ in the same component set, are strictly periodically isotopic in the sense that there is a continuous path of linear periodic nets between them, each of which has the same periodic structure basis, namely 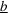. From this we may deduce that there are at most five periodic isotopy classes of embedded nets 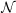 which have the specific LQG (*H*, λ) for some periodic structure. Conceivably there could be fewer periodic isotopy classes since we have not contemplated isotopy paths of nets, with associated paths of periodicity bases, for which the LQG changes several times before returning to (*H*, λ).

Let us also note the following incidental facts about the nets 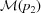. They are 6-coordinated periodic nets and so provide examples of critically coordinated bar-joint frameworks, of interest in rigidity theory and the analysis of rigid unit modes. This is also true of course for all frameworks with the same underlying quotient graph.

## 4. Adjacency depth and model nets   

We now define the adjacency depth of a linear 3-periodic net 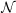. This positive integer can serve as a useful taxonomic index and in Sections 9, 10 we determine, in the case of some small quotient graphs, the 3-periodic graphs which possess an embedding as a (proper) linear 3-periodic net with depth 1. These identifications also serve as a starting point for the determination of the periodic isotopy types of more general depth-1 embedded nets.

We first review maximal periodicity lattices for embedded nets 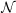 and their primitive periodicity bases.

### 4.1. Primitive periodic structure   

Let 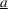 be a vector space basis for 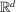 which consists of a periodicity basis for a linear *d*-periodic net 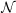. The associated translation group 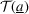 of isometries of 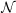 is a subgroup of the space group of 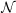. We say that 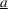 is a primitive, or a maximal periodicity basis, if there is no periodicity basis 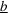 such that 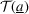 is a proper subset of 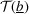.

We focus on 3D and in order to distinguish mirror-related nets we generally consider right-handed periodicity bases of the embedded nets 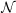.

The next well-known lemma shows that different right-handed primitive bases are simply related by the matrix of an invertible transformation with integer entries and determinant 1. Let 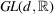 be the group of invertible *d* × *d* real matrices, viewed also as linear transformations of 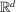, and let 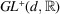 be the subgroup of matrices with positive determinant. Also, let 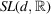 be the subgroup of elements with determinant 1, and 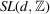 the subgroup of 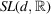 with integer entries.

**Lemma 4.1**

Let 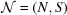 be a linear 3-periodic net in 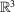 with a primitive right-handed periodicity basis 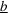 and a right-handed periodicity basis 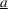. Then 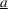 is primitive if and only if there is a matrix 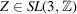 with 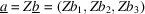.

### The adjacency depth 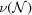 of a linear periodic net   

4.2.

While certain elementary linear periodic nets 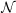 have ‘natural’ primitive periodicity bases, it follows from Lemma 4.1[Statement lemma4.1] that such a basis is not determined by 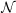. It is natural then to seek a preferred basis 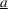 which is a ‘good fit’ in some sense. The next definition provides one such sense, namely that the primitive basis 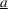 should be one that minimizes the adjacency depth of the pair 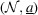.

**Definition 4.2**

The adjacency depth of the pair 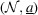, denoted 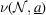, is the depth of the LQG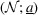, that is the maximum modulus of its edge labels. The adjacency depth, or depth, of 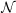 is the minimum value, 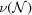, of the adjacency depths 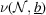 taken over all right-handed primitive periodicity bases 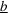.

Let 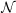 be a linear 3-periodic net with periodicity basis 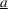. Consider the semi-open parallelepipeds (rhomboids)
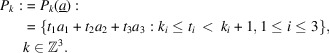
These sets form a partition of 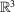, with *P*_*k*_ viewed as a unit cell with label *k*. Note that each cell *P*_*j*_ has 26 ‘neighbours’, given by those cells *P*_*l*_ whose closures intersect the closure of *P*_*k*_. (For diagonal neighbours this intersection is a single point.) Thus we have the equivalent geometric description that 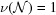 if and only if there is a primitive periodicity basis such that the pair of end nodes of every edge lie in neighbouring cells of the cell partition, where here we also view each cell as a neighbour of itself.

It should not be surprising that for the connected embedded periodic nets of materials the adjacency depth is generally 1. Indeed, while the maximum symmetry embedding 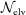 for the net **elv** has adjacency depth 2, it appears to us to be the only connected example in the current RCSR listing with 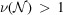. The periodic net **elv** gets its name from the fact that its minimal edge cycles have length 11. On the other hand, in Section 8 we shall see simple examples of multicomponent nets with adjacency depth equal to the number of connected components.

**Definition 4.3**

Let 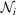 = 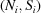, *i* = 1, 2, be linear 3-periodic nets in 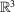. Then 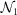 and 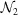 are affinely equivalent [respectively, orientedly (or chirally) affinely equivalent] if there are translates of 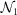 and 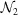 which are conjugate by a matrix *X* in 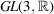 [respectively, 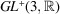].

It follows from the definitions that if 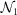 and 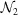 are affinely equivalent then they have the same adjacency depth.

The next elementary lemma is a consequence of the fact that linear 3-periodic nets are, by assumption, proper in the sense that their edges must be noncrossing (*i.e*. essentially disjoint).

**Lemma 4.4**

Let 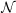 be a linear 3-periodic net with a depth-1 LQG (*H*, λ). Then there are at most seven loop edges on each vertex of *H* and the multiplicity of edges between each pair of vertices is at most 8.

**Proof**

Let 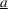 be a periodic structure basis such that 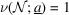. Without loss of generality we may assume that 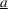 is an orthonormal basis. Let *p*_1_ be a node of 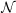. Let *p*_2_, …, *p*_8_ be the nodes *T*_*k*_ *p*_1_ where *k* ≠ (0, 0, 0) with coordinates equal to 0 or 1, and let *p*_9_, …, *p*_27_ be the nodes *T*_*k*_ *p*_1_ for the remaining values of *k* with coordinates equal to 0, 1 or −1. Every line segment [*p*_1_, *p*_*t*_] with *t* ≥ 9 has a lattice translate which either coincides with or intersects, at midpoints, one of the line segments [*p*_1_, *p*_*t*_] with *t* < 9. Since 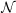 has no edge crossings it follows that there are at most seven translation classes for the edges associated with multiple loops of *H* at a vertex.

We may assume that *p*_1_ = (0, 0, 0). Let *q*_1_ be a node in (0, 1)^3^ in a distinct translation class. Since the depth is 1 it follows that the edges [*q*_1_, *p*] in 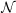 with *p* a translate of *p*_1_, correspond to the positions *p* = λ, where 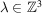 has coordinates taking the values −1, 0 or 1. The possible values of λ are also the labels in the quotient graph of 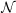 for the edges directed from the orbit vertex of *p*_1_ to the orbit vertex of *q*_1_. There are thus 27 possibilities for the edges [*q*_1_, *p*], and we denote the terminal nodes *p* by λ_*a*_, λ_*b*_, ….

Since 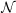 is a proper net, with no crossing edges, we have the constraint that *k* = (λ_*a*_ − λ_*b*_)/2 is not a lattice point for any pair λ_*a*_, λ_*b*_. For otherwise [*q*_1_ + *k*, λ_*b*_ + *k*] is an edge of 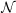 and its midpoint coincides with the midpoint of [*q*_1_, λ_*a*_]. It follows from the constraint that there are at most eight terminal nodes.

□

The following proposition gives a necessary condition for a general 3-periodic graph (*G*, *T*) to have an embedding as a proper linear 3-periodic net. Moreover this condition is useful later for the computational determination of possible topologies for nets which have a quotient graph with one or two vertices.

We say that a LQG (*H*, λ) has the divisibility property, or is divisible, if for some pair of labelled edges (*v*_1_ *v*_2_, *k*), (*v*_1_ *v*_2_, *l*), with the same vertices, and possibly *v*_1_ = *v*_2_, the vector *k* − *l* is divisible in the sense that it is equal to *nt*, with 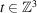 and *n* ≥ 2 an integer. If this does not hold then the three entries of *k* − *l* are coprime and (*H*, λ) is said to be indivisible.

**Proposition 4.5**

Let 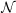 be a (proper) linear 3-periodic net in 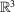 and let (*H*, λ) be a LQG associated with some periodic structure basis for 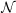. Then (*H*, λ) is indivisible.

**Proof**

Let (*v*_1_ *v*_2_, *k*), (*v*_1_ *v*_2_, *l*) be two edges of (*H*, λ), with *v*_1_ ≠ *v*_2_. Then 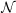 has the incident edges [(*p*(*v*_1_), 0), (*p*(*v*_2_), *k*)], [(*p*(*v*_1_), 0), (*p*(*v*_2_), *l*)] which, by the properness of 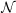, are not collinear. Without loss of generality and to simplify notation assume that the periodicity basis defining the LQG is the standard orthonormal basis. Then these edges are [*p*(*v*_1_), *p*(*v*_2_) + *k*] and [*p*(*v*_1_), *p*(*v*_2_) + *l*]. Taking all translates of these two edges by integer multiples of *t* = *k* − *l* we obtain a 1-periodic (zigzag) subnet, 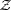 say, of 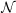 with period vector *t* = (*t*_1_, *t*_2_, *t*_3_).

Suppose next that *t* is divisible with 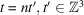 and *n* ≥ 2. Since 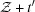 does not coincide with 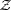 there are crossing edges, a contradiction.

Consider now two loop edges (*v*_1_ *v*_1_, *k*), (*v*_1_ *v*_1_, *l*) and corresponding incident edges in 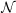, say [*p*(*v*_1_), *p*(*v*_1_) + *k*] and [*p*(*v*_1_), *p*(*v*_1_) + *l*]. Taking all translates of these two edges by the integer combinations *n*_1_ *k* + *n*_2_ *l*, with 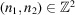, we obtain a 2-periodic subnet, with period vectors {*k*, *l*}, which is an embedding of **sql**. The vector *t* = *k* − *l* is a diagonal vector for the parallelograms of this subnet and so, as before, *t* cannot be divisible.

□

As a consequence of the proof we also see that an embedded net is improper if either of the following conditions fails to hold: (i) for pairs of loop edges in the LQG with the same vertex the two labels generate a maximal rank-2 subgroup of the translation group, (ii) for pairs of nonloop edges the difference of the two labels generates a maximal rank-1 subgroup.

### 4.3. Model nets and LQGs   

We first note that every abstract 3-periodic graph (*G*, *T*) can be represented by a model net 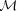 in 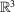 with standard periodicity basis 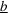, in the sense that *G* is isomorphic to the structure graph 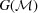 of 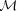 by an isomorphism which induces a representation of *T* as the translation group of 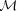 associated with 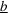. Formally, we define a model net to be such a pair 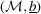 but we generally take the basis choice as understood and use notation such as 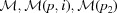 *etc*.

Let (*G*, *T*) be a 3-periodic graph with periodic structure *T* and let *H* = *G*/*T* be the quotient graph (*V*(*H*), *E*(*H*)) determined by *T*. Identify the automorphism group *T* with the integer translation group of 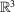. This is achieved through the choice of a group isomorphism 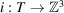 and this choice introduces an ordered triple of generators and coordinates for *T*. Any other such map, *j* say, has the form 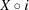 where 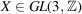.

Label the vertices of *G* by pairs (*v*_*k*_, *g*) where *g* ∈ *T* and *v*_1_, …, *v*_*n*_ is a complete set of representatives for the *T*-orbits of vertices. For the sake of economy we also label the vertices of *H* by *v*_1_, …, *v*_*n*_. Let *p*_*H*_ : *V*(*H*) → [0, 1)^3^ be any injective placement map. Then there is a unique injective placement map 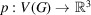 induced by *p* and *i*, with
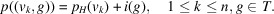
Thus the maps *p*_*H*_, *i* determine a (possibly improper) model embedded net for (*G*, *T*) which we denote as 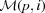. In particular if ((*v*_*k*_, *g*), (*v*_*l*_, *h*)) is an edge of *G* then this determines the line segment edge [*p*((*v*_*k*_, *g*)), *p*((*v*_*l*_, *h*))] of 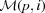. This net is possibly improper since some edges may intersect. Write 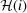 for the LQG (*H*, λ) of 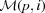 with respect to 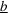. As the notation implies, this depends only on the choice of *i* which coordinatizes the group *T*.

With *i* fixed we can consider continuous paths of such placements, say 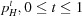, which in turn induce paths of model nets, 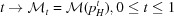. (See also Section 3.1.) When there are no edge collisions, that is, when all the nets 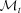 in the path are proper, this provides a strict periodic isotopy between the the pairs 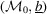 and 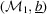 and their given periodic structure bases, 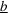. (Such isotopy is also formally defined in the remarks following Definition 6.1[Statement definition6.1].)

Note that if *H* is a bouquet graph, that is, has a single vertex, then the strict periodic isotopy determined by 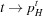 between two model nets for 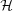 corresponds simply to a path of translations.

In the next proposition we consider 3-periodic graphs as pairs (*G*, *T*), as in Definition 2.5[Statement definition2.5] [and Definition 4.2 of Eon (2011 ▸)]. Moreover we have the following natural notion of isomorphism.

**Definition 4.6**

The pairs (*G*, *T*), (*G*′, *T*′) are isomorphic as 3-periodic graphs if and only if there is a countable graph isomorphism *G* → *G*′ induced by a bijection γ : *V* → *V*′, together with a group isomorphism π : *T* → *T*′ such that γ(*g*(*v*)) = π(*g*)(γ(*v*)) for *v* ∈ *V*, *g* ∈ *T*.

It is in this sense that we may say that an isomorphism (*G*, *T*) → (*G*′, *T*′) of periodic graphs is a pair of isomorphisms (γ, π) which respects the periodic structure.

Note, for example, that the countable structure graph 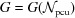 has periodic structure *T* (respectively, *T*′) determined by the periodicity basis (2, 0, 0), (0, 2, 0), (0, 0, 1) [respectively, (4, 0, 0), (0, 1, 0), (0, 0, 1)] for 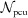. The periodic graphs (*G*, *T*) and (*G*, *T*′) fail to be isomorphic since they have different quotient graphs, which is a necessary condition for this.

**Proposition 4.7**

Let (*G*, *T*), (*G*′, *T*′) be 3-periodic graphs (with given periodic structures) with LQGs 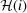 = 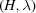, 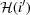 = 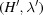 arising from group isomorphisms 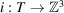 and 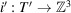. Then the following statements are equivalent.

(i) (*G*, *T*) and (*G*′, *T*′) are isomorphic as 3-periodic graphs.

(ii) There is a graph isomorphism ϕ : *H* → *H*′ and 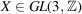 with 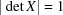 such that λ′(ϕ(*e*)) = *X*(λ(*e*)) for all directed edges *e* of 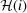.

**Proof**

(ii) 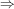 (i). A typical edge *e* of 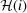 is denoted by a triple [*v*_*e*_, *w*_*e*_, λ(*e*)] and a typical associated edge of *G* is
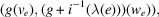
where *g* ∈ *T* and where we have written the group operation in *T* additively. Define γ : *V*(*G*) → *V*(*G*′) by γ(*g*(*v*)) = π(*g*)(ϕ(*v*)) where *v* ∈ *V*(*H*), *g* ∈ *T* and π is the group isomorphism *T* → *T*′ defined by π = (*i*′)^−1^ 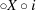. Then γ is a bijection between the vertex sets of *G* and *G*′. Moreover, we note that since 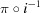 is equal to (*i*′)^−1^ 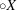 the γ-induced edge [γ(*g*(*v*_*e*_), γ(*g* + *i* ^−1^(λ(*e*)))(*w*_*e*_)] is equal to
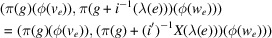
and so is an edge of *G*′, since *X*(λ(*e*)) is equal to λ′(ϕ(*e*)), the label of the edge ϕ(*e*) in 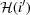. Thus γ induces a graph isomorphism *G* → *G*′ and moreover the pair γ, π is an isomorphism of the periodic graphs, as required.

(i) 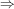 (ii). Consider an isomorphism from (*G*, *T*) to (*G*′, *T*′) given by the pair γ, π. Note that γ_*v*_ : *V*(*G*) → *V*(*G*′) maps *T*-orbits to *T*′-orbits, as does γ_*e*_ : *E*(*G*) → *E*(*G*′), and so γ defines a graph isomorphism ϕ from *H* = *G*/*T* to *H*′ = *G*′/*T*′. Also, the edge [*v*_*e*_, *i* ^−1^(λ(*e*))(*w*_*e*_)] in *G* [associated with the edge *e* = (*v*_*e*_, *w*_*e*_, λ(*e*)) as before] maps to
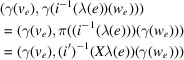
where *X* is the matrix in 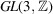 with *X* = 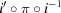. This implies that *X*λ(*e*) must be the label for the associated edge ϕ(*e*) in 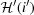, and so (ii) holds.

□

In the case when *H* = *G*/*T* and *H*′ = *G*/*T*′ are bouquet graphs one can say much more. Any graph isomorphism γ : *G* → *G*′ lifts to a linear isomorphism between the model nets 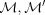 determined by any pair *T*, *T*′ of maximal periodic structures. See for example Proposition 3 of Kostousov (2007 ▸). It follows that for bouquet quotient graph nets we have the following stronger theorem.

**Theorem 4.8**

Let 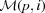 and 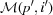 be model nets, with nodes on the integer lattice, for 3-periodic graphs (*G*, *T*) and (*G*, *T*′) with bouquet quotient graphs. Then the following are equivalent. (i) *G* and *G*′ are isomorphic as countable graphs. (ii) 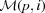 and 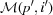 are affinely equivalent by a matrix *X* in 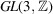.

**Definition 4.9**

A (proper) linear 3-periodic net 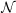 is a lattice net if its set of nodes is a lattice in 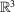.

Equivalently 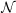 is a lattice net if its quotient graph is a bouquet graph. One may also define a general lattice net in 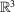 as a (not necessarily proper) embedded net whose quotient graph is a bouquet graph. Theorem 4.8[Statement theorem4.8] shows that lattice nets (even general ones) are classified up to affine equivalence by their topologies. In Theorem 9.5[Statement theorem9.5] we obtain a proof of this in the depth-1 case through a case-by-case analysis. Also we show that for the connected depth-1 lattice nets there are 19 classes.

In principle Proposition 4.7[Statement proposition4.7] could be used as a basis for a computational classification of periodic nets with small quotient graphs with a depth-1 labelling. However we note that there are more practical filtering methods such as those underlying Proposition 10.1[Statement proposition10.1] which determines the 117 connected topologies associated with certain depth-1 nets which are supported on two parallel vertex lattices in a bipartite manner.

## 5. Linear graph knots   

Let *H* be a multigraph, that is, a general finite graph, possibly with loops and with an arbitrary multiplicity of ‘parallel’ edges between any pair of vertices. Then a graph knot in 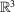 is a faithful geometric representation of *H* where the vertices *v* are represented as distinct points *p*(*v*) in 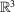 and each edge *e* with vertices *v*, *w* is represented by a smooth path 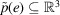, with endpoints *p*(*v*), *p*(*w*). Such paths are required to be free from self-intersections and disjoint from each other, except possibly for coinciding endpoints. Thus a graph knot *K* is formally a triple 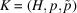, and we may also refer to this triple as a spatial graph or as a proper placement of *H* in 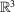. It is natural also to denote a graph knot *K* simply as a pair (*N*, *S*), where *N* is the set of vertices, or nodes, *p*(*v*) in 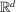, and *S* is the set of nonintersecting paths 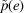. We remark that spatial graphs feature in the mathematical theory of intrinsically linked connected graphs (Conway & Gordon, 1983 ▸; Kohara & Suzuki, 1992 ▸).

One can similarly define a graph knot *K* in any smooth manifold and of particular relevance is the Riemannian manifold known as a flat 3-torus. This is essentially the topological 3-torus identified naturally with the set [0, 1)^3^ and the topology, in the usual mathematical sense, is the natural one associated with continuity of the quotient map 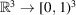. Moreover we define a line segment in the flat torus to be the image of a line segment in 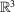 under this quotient map. The curiosity here is that such a flat torus line segment may appear as the union of several line segment sets in [0, 1)^3^.

We formally define a linear graph knot in the flat torus to be a triple 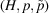, or a pair (*N*, *S*), where the vertices, or nodes, *p*(*v*) lie in [0, 1)^3^ and the paths, or edges, 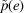, are essentially disjoint flat torus line segments. Intuitively, this is simply a finite net in the flat 3-torus with linear nonintersecting edges.

We now associate a linear graph knot *K* in [0, 1)^3^ with an embedded net 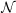 with a specified periodicity basis 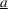. Informally, this is done by replacing 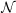 by its affine normalization 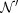, wherein 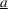 is rescaled to the standard basis, and defining *K* as the intersection of the body 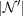 with [0, 1)^3^. That is, one takes the simplest model net 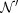 for 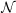 and ignores everything outside the cube [0, 1)^3^.

For the formal definition, let (*F*_*v*_, *F*_*e*_) be a motif for 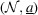, where 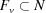 (respectively, 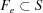) is a finite set of representatives for translation classes of nodes (respectively, edges) of 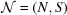, with respect to 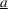. Let 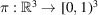 be the natural quotient map associated with the ordered basis 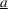. This is a composition of the linear map for which 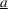 maps to the standard right-handed basis, followed by the quotient map. Define *p* : *F*_*v*_ → [0, 1)^3^ to be the induced injection and 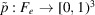 to be the induced map from closed line segments to closed line segments of the flat torus [0, 1)^3^.

**Definition 5.1**

Let *H* be the quotient graph for the pair 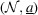. The triple 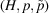 is the linear graph knot of 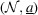 and is denoted as 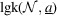.

Since 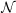 is necessarily proper, with essentially disjoint edges, the placement 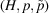 has essentially disjoint edges and so is a linear graph knot.

Note that the linear graph knot determines uniquely the net 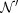 which in fact can be viewed as its covering net. It follows immediately that if 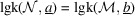 then 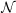 and 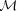 are linear periodic nets which are orientedly affine equivalent.

We now give some simple examples together with perspective illustrations. Such illustrations are unique up to translations within the flat 3-torus and so it is always possible to arrange that the nodes are interior to the open unit cube. In this case the 3D diagram reveals their valencies. On the other hand, as we saw in the partial body examples in Section 3.1 it can be natural to normalize and simplify the depiction by a translation which moves a node to the origin.

**Example 5.2**

**Figure 3:**
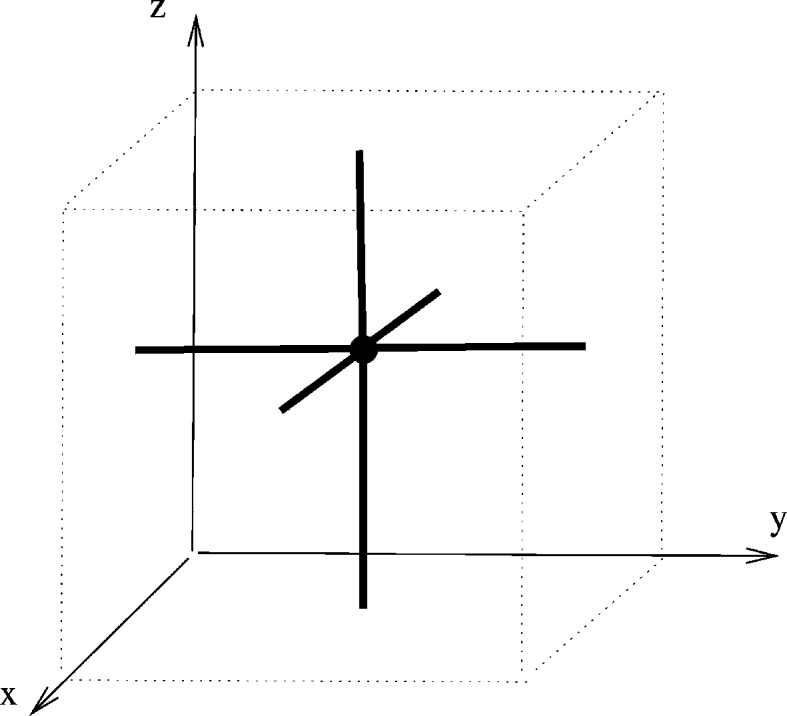
The linear graph knot *K*_pcu_ on the flat 3-torus [0, 1)^3^.

The simplest proper linear 3-periodic net is the primitive cubic net 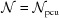. We may normalize this so that the node set is a translate of the set 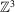. The standard primitive periodic structure basis gives the graph knot 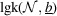, which we denote as *K*_pcu_ and which is illustrated in Fig. 3 ▸. The three ‘line segment’ edges in the flat torus are here depicted by three pairs of line segments. The quotient graph of 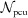, which is also the underlying graph of *K*_pcu_, has one vertex and three loop edges. Note that if the node is translated to the origin then the depiction of the loop edges is given by three axial line segments.

By taking a union of *n* disjoint generic translates (within [0, 1)^3^) of *K*_pcu_ one obtains the linear graph knot of an associated multicomponent linear net. In Theorem 8.2[Statement theorem8.2] we compute the number of periodic isotopy classes of such nets and the graph knot perspective is helpful for the proof of this.

**Example 5.3**

**Figure 4:**
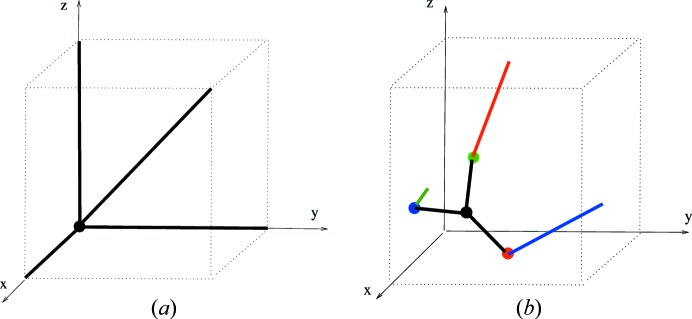
Linear graph knots on the flat torus for (*a*) **bcu** and (*b*) **srs**.

Fig. 4 ▸ shows linear graph knots (or finite linear nets) on the flat torus for the maximal symmetry nets 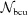 and 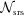. Each is determined by a natural primitive right-handed depth-1 periodicity basis 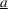 which, by the definition of 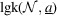, is normalized to 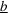. The quotient graphs for these examples are, respectively, the bouquet graph with four loop edges and the complete graph on four vertices. The periodic extensions of these graph knots give well-defined model nets, say 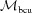 and 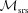, which are orientedly affinely equivalent to the maximal symmetry nets 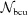 and 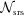.

**Example 5.4**

The linear 3-periodic net 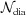 for the diamond crystal net (with maximal symmetry) has a periodic structure basis 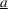 corresponding to three incident edges of a regular tetrahedron, and has a motif consisting of two vertices and four edges. The graph knot 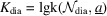 is obtained by (i) an oriented affine equivalence with a model net 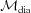 with standard orthonormal periodic structure basis, and (ii) the intersection of 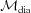 with [0, 1)^3^. This graph knot has an underlying graph *H*(0, 4, 0) (in the notation of Section 10) with two vertices and four nonloop edges.

**Figure 5:**
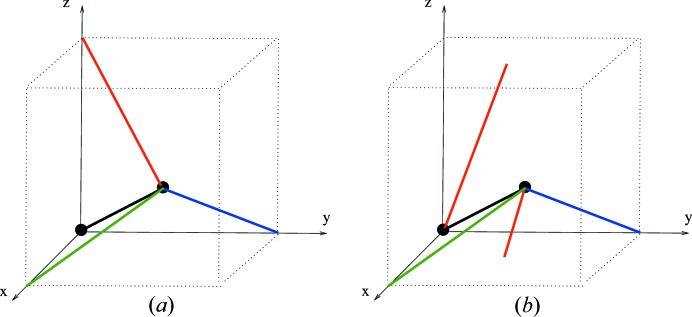
Linear graph knots (*a*) *K*_1_ and (*b*) *K*_2_ associated with **dia**.

In Figs. 5 ▸, 6 ▸ we indicate four graph knots which define model nets each with underlying net (structure graph) equal to **dia**. In fact the graph knots *K*_1_, *K*_2_ are rotationally linearly isotopic (see Definition 6.3[Statement definition6.3]). To see this consider a linear graph knot homotopy starting with *K*_1_ which is determined by a downward motion of the central vertex [at (1/2, 1/2, 1/2) say] of *K*_2_ through the floor of [0, 1)^3^. The edge deformations are determined and, since the floor is equal to the roof, we can terminate the vertex motion at (1/2, 1/2, 1/2). Note that there are no edge crossings, so that the homotopy is in fact an isotopy. Moreover, examining the edges, one of which is re-entrant, we see that the final linear graph knot is equal to the image of *K*_1_ under a half-turn rotation about the line through (1/2, 1/2, 1/2) and (0, 0, 1/2).

On the other hand *K*_2_ and *K*_3_ are linearly isotopic in terms of a motion of the vertex of *K*_2_ at the origin to the position of the left-hand vertex of *K*_3_. It follows from this that the associated model nets 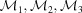 are strictly periodically isotopic, simply by taking the periodic extension of these isotopies to define periodic isotopies.

In contrast to this, observe first that the linear graph knot *K*_4_ is obtained from *K*_3_ by a continuous motion of the nodes *p*_1_ and *p*_2_ to their new positions in the 3-torus. Such a motion defines a linear homotopy in the natural sense. The (uniquely) determined edges of the intermediate knots in this case inevitably cross at some point in the motion so these linear homotopies are not linear isotopies. The model net 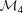 for the knot *K*_4_ is in fact not periodically isotopic to the unique maximal symmetry embedding 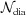, and so is self-entangled. We show this in Example 6.7[Statement example6.7].

In the model nets of the examples above we have taken a primitive periodic structure basis with minimal adjacency depth. In view of this the represented edges between adjacent nodes in these examples have at most two diagramatic components, that is they reenter the cube at most once. In general the linear graph knot associated with a periodic structure basis of depth 1 has edges which can reenter at most three times.

**Remark 5.5**

**Figure 6:**
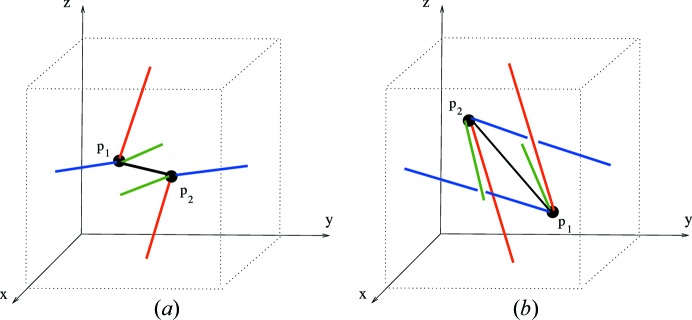
Linear graph knots (*a*) *K*_3_ and (*b*) *K*_4_ associated with **dia**.

We shall consider families of embedded nets up to oriented affine equivalence and up to periodic isotopy. In general there may exist enantiomorphic pairs, that is, mirror images 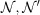 which are not equivalent. This is the case, for example, for embeddings of **srs**. However, such inequivalent pairs do not exist if the quotient graph is a single vertex (lattice nets) or a pair of vertices with no loop edges (double lattice nets with bipartite structure). This becomes evident in the latter case, for example, on considering an affine equivalence with a model net for which the point (1/2, 1/2, 1/2) is the midpoint of the two representative nodes in the unit cell. This midpoint serves as a point of inversion for the model net (or, equivalently, its graph knot). The graph knots in Fig. 6 ▸ indicate such centred positions.

**Remark 5.6**

We have observed that for a linear 3-periodic net the primitive right-handed periodicity bases 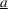 are determined up to transformations by matrices in 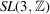. Such matrices induce chiral automorphisms of the flat 3-torus which preserve the linear structure. Accordingly (and echoing the terminology for embedded nets) it is natural to define two graph knots on the same flat torus to be orientedly (or chirally) affinely equivalent if they have translates which correspond to each other under such an automorphism. Thus, to each linear 3-periodic net 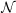 one could associate its primitive graph knot, on the understanding that it is only determined up to oriented affine equivalence.

**Remark 5.7**

We remark that triply periodic surfaces may be viewed as periodic extensions of compact surfaces on the flat 3-torus. It follows that the tilings and triangulations of these compact surfaces generate special classes of linear 3-periodic nets. Such nets have been considered, for example, in the context of periodic hyperbolic surfaces and minimal surfaces, where the methods of hyperbolic geometry play a role in the definition of isotopy classes (Evans *et al.*, 2013 ▸; Hyde *et al.*, 2003 ▸; see also Hyde & Delgado-Friedrichs, 2011 ▸).

## 6. Isotopy equivalence   

Consider the following informal question: when can 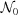 be deformed into 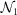 by a continuous path with no edge crossings?

This question is not straightforward to approach for two reasons. Firstly, a linear periodic net may contain, as a finite subnet, a linear realization of an arbitrary knot or link. For example, the components of 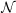 could be translates of a linear realization of an arbitrary finite knot where all vertices have degree 2. (Here 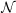 would have dimension type {3; 0}.) Thus, resolving the question by means of discriminating invariants is in general as hard a task as the corresponding one for knots and links. Secondly, the rules for such deformation equivalence need to be decided upon, and, *a priori*, the deformation equivalence classes are dependent on these rules.

The following definition may be regarded as the natural form of isotopy equivalence appropriate for the category of embedded periodic nets in 3D which have line segment bonds, no crossing edges and no coincidences of node locations (node collisions).

**Definition 6.1**

Let 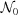 and 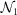 be proper linear 3-periodic nets in 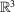. Then 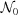 and 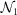 are periodically isotopic, or have the same periodic isotopy type, if there is a family of such (noncrossing) nets, 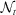, for 0 < *t* < 1, for which

(*a*) there is a continuous path of bases of 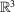, 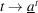, 0 ≤ *t* ≤ 1, where 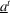 is a right-handed periodicity basis for 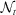,

(*b*) there are bijective functions 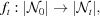 for 0 ≤ *t* ≤ 1, which map nodes to nodes, such that

(i) *f*_0_ is the identity map on 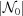,

(ii) for each node point *p* in 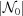 the map *t* → *f*_*t*_(*p*) is continuous,

(iii) the restriction of *f*_*t*_ to each edge [*a*, *b*] is the unique affine map onto the image edge, [*f*_*t*_(*a*), *f*_*t*_(*b*)] in 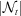 determined by linear interpolation.

We make a number of immediate observations:

(1) The condition (iii) could be omitted but is a conceptual convenience in that it implies that each map *f*_*t*_ from the body of 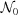 to the body of 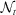 is determined by its restriction to the nodes.

(2) The definition applies to entangled nets with several connected components and in this case the isotopy can be viewed as a set of *n* independent periodic isotopies, for the *n* components, with the same time parameter *t* and periodicity bases 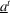, and subject only to the noncollision of components for each value of *t*.

(3) Every such net 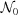 is periodically isotopic to a model net 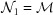 with periodicity basis 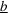. Indeed, for any right-handed periodicity basis 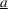 for 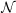 there is an elementary isotopy equivalence from 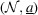 to a unique pair 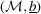 which is determined by a path of transformations from 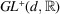 which in turn is determined by any continuous path of bases from 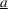 to 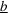.

(4) If 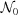 and 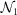 are orientedly affine equivalent then they are periodically isotopic since the topological group 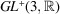 is path-connected.

We also define the pair 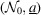 to be strictly periodically isotopic to the pair 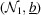 if there is an isotopy equivalence 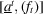, as in parts (*a*), (*b*) of the definition with 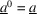 and 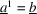. In view of the previous observations we have the following:

*Equivalent definition*. The embedded periodic nets 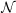 and 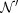 in 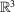 are periodically isotopic if there is a rescaling and rotation of 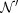 to a net 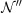 so that (i) 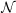 and 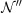 have a common embedded translation group with basis 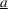, and (ii) 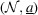 and 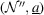 are strictly periodic isotopic.

Strict periodic isotopy is evidently an equivalence relation on the set of pairs 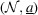. Periodic isotopy is also an equivalence relation but this is not so immediate. However, as the next proof shows, one can replace a pair of given periodic isotopies, between 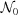 and 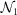 and between 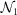 and 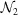, by a new pair such that the paths of periodicity bases can be concatenated, and so provide an isotopy between 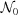 and 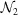.

**Theorem 6.2**

Periodic isotopy equivalence is an equivalence relation on the set of proper linear 3-periodic nets.

**Proof**

Let 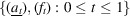 be an isotopy equivalence for 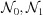 as above and let 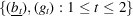 be an isotopy equivalence between 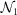 and 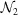. Suppressing the implementing maps *f*_*t*_ and *g*_*t*_ we may denote this information as
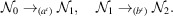
We now have two periodic structures 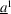 and 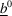 on 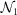. If they were the same then a periodic isotopy between 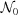 and 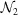 could be completed by the simple concatenation of these paths. However, in general we must choose new periodic structures to achieve this.

For a periodic structure basis 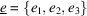 and 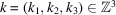 let us write 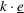 for {*k*_1_ *e*_1_, *k*_2_ *e*_2_, *k*_3_ *e*_3_}. We have 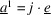 for some primitive periodic structure basis 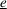 of 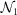. Similarly 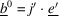 for some primitive periodic structure basis 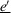. Since primitive right-handed periodicity bases on the same linear periodic net are equivalent by a linear map 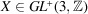, it follows that the vectors of 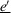 are integral linear combinations of the vectors of 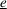. Thus the vectors of 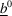 are integral linear combinations of the vectors of 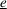. It follows that we can now find elements 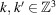 so that the vectors of 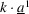 are integral linear combinations of the vectors of 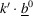.

Consider now the induced isotopy equivalences
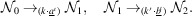
These isotopies are identical to the previous isotopy equivalences at the level of the paths of individual nodes, but the framing periodic structure bases have been replaced. These periodic isotopies do not yet match, so to speak, but we note that the second isotopy equivalence implies an isotopy equivalence from 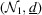 to some 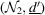 whenever the periodic structure basis 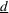 has vectors which are integer combinations of the vectors of 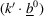. Thus we can do this in the case 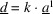 to obtain matching isotopy paths, in the sense that the terminal and initial periodic structure bases on 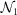 agree. Composing these paths we obtain the desired isotopy equivalence between 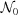 and 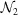.

□

### 6.1. Isotopy equivalence for linear graph knots   

In the next definition we formally define two linear graph knots on the flat torus to be linearly isotopic if there is a continuous path of linear graph knots between them. It follows that if the linear graph knots 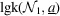 and 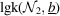 are linearly isotopic then, by simple periodic extension, the nets 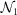 and 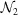 are periodically isotopic. Also we see in Proposition 6.5[Statement proposition6.5] a form of converse, namely that if 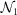 and 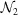 are periodically isotopic then they have graph knots, associated with some choice of periodic structures, which are linearly isotopic.

On the other hand, note that a linear 3-periodic net 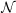 in 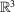 with the standard periodicity basis {*b*} is periodically isotopic to its image 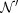 under an isometric map which cyclically permutes the coordinate axes. This is because there is a continuous path of rotation maps of 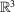 from the identity map to the cyclic rotation and restricting these maps to 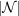 provides maps (*f*_*t*_) for a periodic isotopy. While the associated graph knots 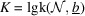 and 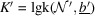, considered as knots in the same 3-torus, are homeomorphic (under a cyclic automorphism of the 3-torus which maps one graph knot to the other) they need not be linearly isotopic. This follows since linear isotopy within a fixed 3-torus must preserve the homology classes of cycles and yet *K* may contain a directed cycle of edges with a homology class in 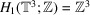 which do not appear as a homology class of any cycle of edges in *K*′.

In view of this, in the next formal definition we also give weaker forms of linear isotopy equivalence which can be considered as linear isotopy up to rotations and linear isotopy up to affine automorphisms.

Let 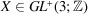. Then there is an induced homeo­morphism of the flat 3-torus which we denote as *X*_π_. This is affine in the sense that flat torus line segments map to flat torus line segments.

**Definition 6.3**

Let *K*_0_ = (*N*_0_, *S*_0_) and *K*_1_ = (*N*_1_, *S*_1_) be linear graph knots on the flat torus 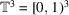.

(i) *K*_0_ and *K*_1_ are linearly isotopic if there are linear graph knots *K*_*t*_ = (*N*_*t*_, *S*_*t*_), for 0 < *t* < 1, and bijective continuous functions *f*_*t*_ : |*K*_0_| → |*K*_*t*_| such that *f*_0_ is the identity map on *K*_0_, *f*_*t*_(*N*_0_) = *N*_*t*_, *f*_*t*_(*S*_0_) = *S*_*t*_, and the paths *t* → *f*_*t*_(*p*), for *p* ∈ *K*_0_ and 0 ≤ *t* ≤ 1, are continuous.

(ii) *K*_0_ and *K*_1_ are rotationally linearly isotopic if for some rotation automorphism *X*_π_, with *X* a rotation in 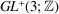, the graph knots *K*_1_ and *X*_π_ *K*_2_ are linearly isotopic.

(ii) *K*_0_ and *K*_1_ are globally linearly isotopic if for some affine automorphism *X*_π_, with 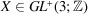, the graph knots *K*_1_ and *X*_π_ *K*_2_ are linearly isotopic.

### 6.2. Enumerating linear graph knots and embedded nets   

We can indicate a linear graph knot *K* on the flat 3-torus by the triple (*Q*, *h*, *p*), where (*Q*, *h*) is a labelled directed quotient graph and *p* = (*x*_1_, …, *x*_*n*_) denotes the positions of its *n* vertices in the flat 3-torus 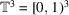. We may also define general placements of *K*, or of (*Q*, *h*), as triples (*Q*, *h*, *p*′) associated with points *p*′ in the *n*-fold direct product 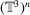. Such placements either correspond to proper linear graph knots with the same LQG, or are what we shall call singular placements, for which the nodes *x*_*i*_′ of *p*′ may coincide, or where some pairs of line segment bonds determined by (*Q*, *h*) and *p*′ are not essentially disjoint.

The general placements of *K* are thus parametrized by the points *x*′ of the flat 3*n*-torus 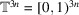, and this manifold is the disjoint union of the set 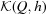 of proper placements and the set 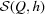 of singular placements.

**Theorem 6.4**

There are finitely many linear isotopy classes of linear graph knots in the flat torus 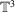 with a given LQG.

The following short but deep proof echoes a proof used by Randell (1998 ▸) in connection with invariants for finite piecewise linear knots in 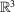. However, we remark that an alternative more intuitive proof of this general finiteness theorem could be based on the fact that the isotopy classes of the linear graph knots can be labelled by finitely many crossing diagrams (appropriate to the 3-torus). Also direct arguments are available to show such finiteness for LQGs with one or two vertices.

**Proof**

It suffices to show that there are finitely many representative linear graph knots (with the same given LQG) so that any linear graph knot (with the given LQG) is linearly isotopic to one of them. The set 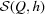 is a closed semi-algebraic set since it is defined by a set of polynomials and inequalities. The open set 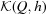 is equal to 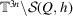. Since this set is the difference of two algebraic sets it follows from the structure of real algebraic varieties (Whitney, 1957 ▸) that the number of connected components of 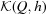 is finite. Taking a representative linear graph knot from each of these components completes the proof.

□

The theorem implies that the isotopy classes of linear graph knots are countable, since LQGs are countable, and so in principle these classes may be listed by various schemes. For example, for each *n* there are finitely many LQGs of depth 1 with *n* vertices and so there are finitely many linear isotopy classes of linear graphs knots with *n* vertices and depth 1.

The corollary of the next elementary proposition gives a similar finiteness for the periodic isotopy classes of embedded periodic nets.

**Proposition 6.5**

Let 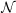 and 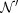 be linear 3-periodic nets in 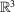. Then the following are equivalent. (i) 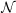 and 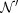 are periodically isotopy equivalent. (ii) There are right-handed periodicity bases 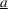 and 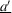 for 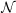 and 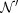 such that the linear graph knots 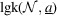 and 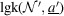 are linearly isotopic.

**Proof**

Suppose that (i) holds. Let 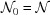 and 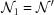 and assume the equivalence is implemented, as in the definition of periodic isotopy, by a path of intermediate nets 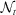 together with (*a*) a continuous path of bases 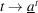, 0 ≤ *t* ≤ 1, where 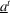 is a periodicity basis for 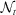, and (*b*) bijective functions *f*_*t*_ from the set of nodes of 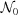 to the set of nodes of 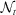. The functions *f*_*t*_ necessarily respect the periodic structure. Let 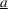 = 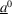 and 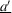 = 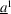. It follows that the resulting path 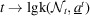 is an isotopy between 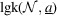 and 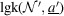.

Suppose that (ii) holds, with 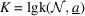 and 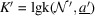. A linear isotopy equivalence (*f*_*t*_) between *K* and *K*′ extends uniquely, by periodic extension, to a periodic isotopy equivalence between 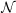 and 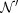.

□

**Corollary 6.6**

Let (*H*, λ) be a LQG. Then there are finitely many periodic isotopy classes of linear 3-periodic nets 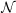 which have the LQG (*H*, λ) with respect to some periodicity basis.

**Proof**

Fix a LQG (*H*, λ). Then a linear 3-periodic net 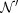 which has the LQG (*H*, λ) with respect to some periodicity basis is periodically isotopic to a linear 3-periodic net 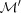 which has LQG (*H*, λ) with respect to the standard basis. It suffices to show that the set of such model nets 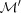 has finitely many periodic isotopy classes. This follows since, by Theorem 6.4[Statement theorem6.4], their linear graph knots (for the standard basis) have finitely many linear isotopy types and (as in the proof of the previous proposition) a linear isotopy at the graph knot level determines a periodic isotopy at the level of nets, simply by periodic extension.

□

In future work it will be of interest to focus on individual topologies and to determine the finitely many periodic isotopy classes of depth 1. Of particular interest are those with some sense of maximal symmetry over their periodic isotopy class. In fact we formalize this idea in Section 7.2 in connection with homogeneous multicomponent nets.

We now note two basic examples of connected self-entangled nets, which we regard as periodic isotopes of their maximal symmetry embedded nets.

**Example 6.7**

**Figure 7:**
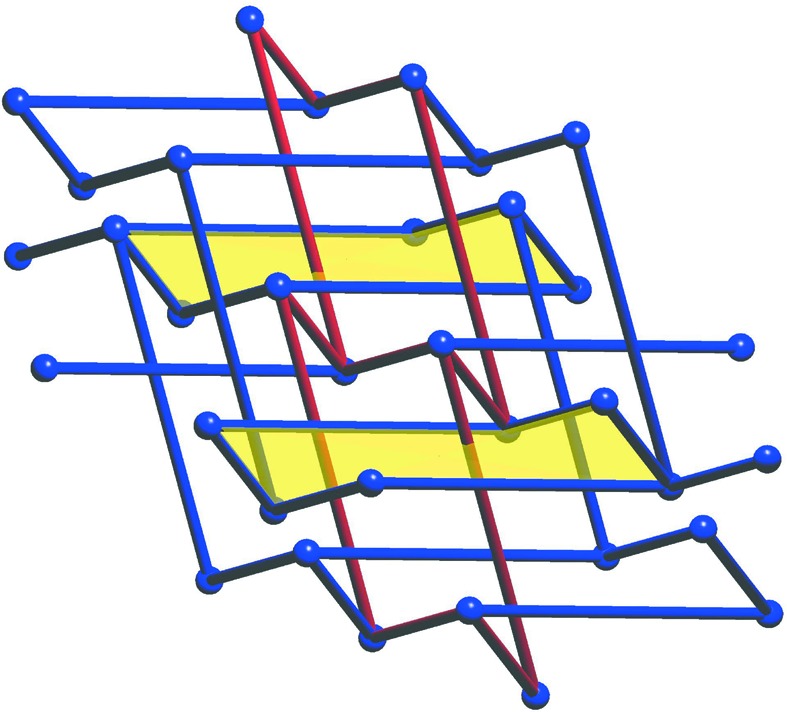
Catenated 6-cycles in a self-entangled embedding of **dia**.

Self-entangled diamond. The multi-node fragment in Fig. 7 ▸ shows part of an embedded net, say 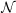, whose topology is **dia**. That 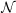 and the usual maximum symmetry net 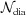 are not periodically isotopic follows from an examination of the catenation of cycles. Specifically the diagram shows that 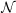 has two disjoint 6-cycles of edges which are linked. This property does not hold for 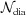 and so they cannot be periodically isotopic.

**Example 6.8**

**Figure 8:**
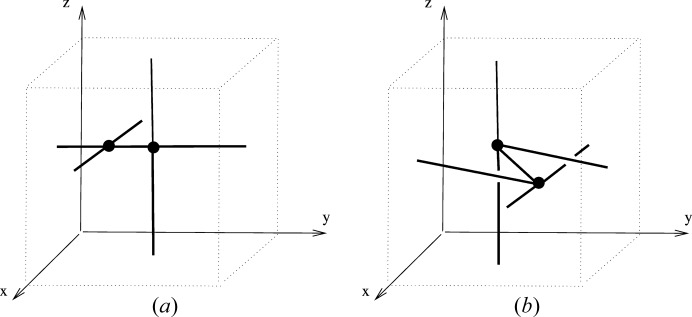
Linear graph knots for distinct periodic isotopes of **cds** for which (*a*) the covering net is not self-entangled and (*b*) the covering net is self-entangled.

Self-entangled embeddings of **cds**. The maximal symmetry net 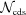 (associated with cadmium sulfate) has an underlying periodic net **cds** with quotient graph *H*(1, 2, 1). The left-hand diagram of Fig. 8 ▸ indicates a linear graph knot for **cds** and the 3-periodic extension of this diagram defines a model embedded net which is periodically isotopic to 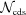. To be precise, define this net as the model net 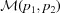 with *p*_1_ = (1/2, 1/2, 1/2), *p*_2_ = (1/2, 1/4, 1/2) and with LQG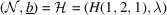 where λ assigns the labels, (0, 0, 1) to the loop edge associated with *p*_1_, (1, 0, 0) to the loop for *p*_2_, and the labels (0, 0, 0) and (0, 1, 0) to the two remaining edges.

As in Section 3.1, let us now view *p*_2_ as variable point 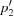 = (*x*, *y*, *z*) within the semi-open cube [0, 1)^3^. The positions of 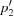 together with the LQG define model nets as long as there are no edge crossings. Let 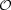 be the set of these positions for 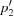. Then, viewed as a subset of [0, 1)^3^ (not as a subset of the flat 3-torus) the set 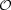 decomposes as the union of five path-connected components 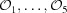. The set 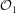 is the subset of 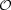 with *y* < 1/2, the set 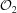 is the subset with *y* > 1/2, *x* > 1/2, *z* < 1/2 (the right-hand figure of Fig. 8 ▸ corresponds to a point in 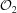), and 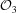 is the subset with *y* > 1/2, *x* < 1/2, *z* < 1/2. The sets 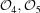 are similarly defined to 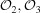, respectively, except that *z* is greater than 1/2.

Let 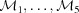 be representatives for the five path-connected components. The net 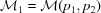 is a model net for 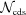 while the net 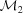 is a periodic isotope. This can be seen once again by the different catenation properties exhibited. Specifically, 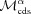 has a 6-cycle of edges which is linked to (penetrated by) an infinite linear subnet, while 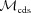 does not have such catenation.

### 6.3. Entangled nets, knottedness and isotopies   

The examples above concern connected self-entangled nets and their connected graph knots on the 3-torus and there is a natural intuitive sense in which such nets can be ‘increasingly knotted’ by moving through homotopies to embeddings with an increasing number of edge crossings. However, the linear graph knot association is also a helpful perspective for multicomponent nets whose components are not self-entangled so may be equal to, or perhaps merely isotopic to, their individual maximal symmetry embeddings. In this case there are intriguing possibilities for the nesting of such ‘unknotted components’ and their associated space groups. We address this topic in Sections 7 and 8 as well as the attendant crystallographic issue of formulating a notion of maximal symmetry for such multicomponent nets.

For completeness we note two further forms of isotopy equivalence which will not be of concern to us.

(i) Relaxed periodic isotopy. The notion of periodic isotopy equivalence in Definition 6.1[Statement definition6.1] can be weakened in a number of ways. One less strict form, which one could call relaxed periodic isotopy, omits the condition (*a*), requiring periodic basis continuity, and so allows a general continuous path of intermediate (noncrossing) periodic nets 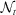. Since the continuity requirement in (*b*) of the node path functions (*f*_*t*_) is one of point-wise continuity on the set of nodes 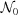, it follows that such paths of periodic embedded nets can connect embedded nets that are not periodically isotopic. In particular, one can construct relaxed periodic isotopies which untwist infinitely twisted components (*e.g.* straightening an entangled double helix to a pair of parallel linear strands).

(ii) Ambient isotopy. The usual definition of ambient isotopy for a pair *K*_1_, *K*_2_ of knots (or links) in 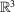 requires the existence of a path *h*_*t*_ of homeomorphisms of 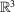 (the ambient space) such that *h*_0_ is the identity map and *h*_1_(*K*_1_) = *K*_2_. Here, for 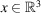, we have *h*_*t*_(*x*) = *h*(*t*, *x*) where 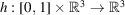 is a continuous function. Also, the closed sets *K*_*t*_ = *h*(*t*, *K*_0_), for 0 ≤ *t* ≤ 1, form a path of knots (or links) between *K*_0_ and *K*_1_.

One may similarly define ambient isotopy for embedded periodic nets (Delgado-Friedrichs & O’Keeffe, 2005 ▸). In this case the intermediate closed sets *L*_*t*_, defined by 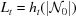, are the bodies of general string-node nets 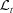. We recall from Power & Schulze (2018 ▸) that a string-node net 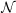 in the Euclidean space 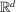 is a pair (*N*, *S*) of sets (whose respective elements are the nodes and strings of 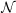) with the following two properties. (*a*) *S* is a nonempty finite or countable set whose elements are lines, closed line segments or closed semi-infinite line segments in 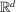, such that collinear strings are disjoint. (*b*) *N* is a nonempty finite or countable set of points in 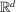 given by the intersection points of strings.

It is natural to impose the further condition that these sets are the bodies of (proper) linear 3-periodic nets, and this then gives a definition of what might be termed locally periodic ambient isotopy. In this case the set of restriction maps 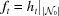 define a (stricter form of) relaxed periodic isotopy.

## 7. Group methods and maximal symmetry isotopes   

We now give some useful group-theoretic perspectives for multicomponent frameworks, starting with the general group–supergroup construction in Baburin (2016 ▸) for transitive nets. This method underlies various algorithms for construction and enumeration. In this direction we also define maximal symmetry periodic isotopes in terms of extremal group–supergroup indices of the components. Finally, turning towards generically, or randomly, nested components, we indicate the role of Burnside’s lemma in counting all periodic isotopes for classes of shift-homogeneous nets.

### 7.1. Group–supergroup constructions   

Let 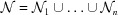 be a linear 3-periodic net which is a disjoint union of connected linear 3-periodic nets in 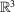. Let *G* be the space group of 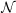 and assume that it acts transitively on the *n* components of 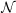. Thus 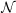 is a transitively homogeneous net, or is of transitive type.

Let *g*_1_ = id, the identity element of *G*, and note that for each *i* = 2, …, *n* there is an element *g*_*i*_ ∈ *G* with 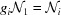. Also, let 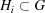 be the subgroup of elements *g* with 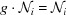, for *i* = 1, …, *n*.

**Lemma 7.1**

The cosets of *H*_1_ in *G* are *g*_1_ *H*_1_, …, *g*_*n*_ *H*_1_.

**Proof**

The cosets *g*_*i*_ *H*_1_ are distinct, since their elements map 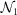 to the distinct subnets 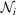. On the other hand, if *g* ∈ *G* then 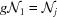 for some *j* and so 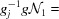 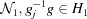, 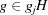 and *gH*_1_ = *g*_*j*_ *H*_1_.

□

Write 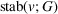 to indicate the subgroup of *G* which fixes the node *v* of 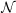 and similarly define the stabilizer group of an edge *e* of 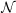.

**Lemma 7.2**

Let *v* (respectively, *e*) be a node (respectively, edge) of 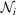 for some *i*. Then
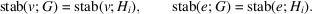

**Proof**

It suffices to show that if *g* fixes an element (vertex or edge) of 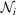 then 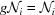. Observe that 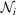 is the maximal connected subnet of 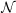 containing the element. Also, for any subnet 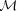 the image 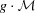 is connected if and only if 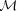 is connected, and so the lemma follows.

□

These lemmas feature in the proof of the following theorem (Baburin, 2016 ▸).

**Theorem 7.3**

If *g* ∈ *G* is a mirror element then *g* ∈ *H*_1_.

The significance of this result is that it shows that the construction of a transitive-type entangled net 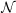 with a connected component 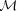 requires the space group 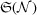 to be free of mirror symmetries which are not in 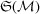. In fact this necessary condition is frequently a sufficient condition and this leads to effective constructions of novel entangled nets where these nets have components 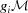 with multiplicity equal to the index of 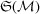 in 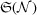.

### 7.2. Maximal symmetry periodic isotopes   

Let 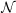 be a multicomponent embedded 3-periodic net in 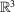 with space group 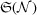 and let 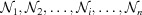 be representatives of the equivalence classes of the components of 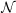 for the translation subgroup of 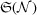. Also, as in the previous section, let *H*_*i*_ be the setwise stabilizer of 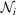 in 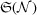. Regarding *H*_*i*_ as a subgroup of 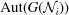 (*cf*. Section 2.2) we may compute the indices 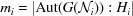. Here we restrict our scope to crystallographic nets 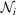 (Klee, 2004 ▸) and therefore the indices are always finite. These indices evidently coincide when 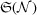 is transitive on the components of 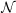 and this is our primary focus.

We say that 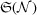 is a maximal symmetry space group for the periodic isotopy class of 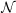 if the nondecreasing rearrangement 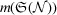 of *m*_1_, …, *m*_*n*_, which we call the multi-index of 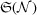, is minimal for the lexicographic order when taken over all groups 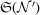 where 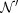 is periodically isotopic to 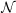. In this case we refer to 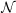 as a maximal symmetry periodic isotope and we write 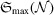 for this space group, noting that 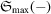 is only defined for such minimal multi-index embedded nets. We note that a maximal symmetry proper embedding of a multicomponent net need not be unique, as might be already the case for (connected) single-component nets (*cf*. Section 2.2).

In the same way one may define maximal symmetry groups for periodic homotopy and one may consider other equivalence relations depending on the matter at hand but these issues shall not concern us here.

We note that a maximal symmetry embedding for periodic isotopy is related to the concept of an ideal geometry of a knot (Evans *et al.*, 2015 ▸, and references therein) that is required to minimize some energy function. However, as well as a certain arbitrariness in the choice of energy function and the possibility of overlooking a global minimum, the result of optimization depends on the imposed periodic boundary conditions. Thus the determination of maximal translational symmetry embeddings remains problematic in the search for an ideal geometry of a multicomponent periodic net. In contrast, our definition, being essentially group-theoretic, aims to capture isotopically intrinsic properties of embedded nets which are independent of such constraints.

Maximizing the symmetry of interpenetrated embedded nets is important for a number of reasons, *e.g.* to characterize their transitivity properties and to derive possible distortions which might occur in a crystal structure by examining group–subgroup relations. Furthermore, the knowledge of a maximal symmetry can be used to explicitly construct a deformation path that relates an embedding with maximal symmetry to a distorted embedding 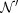 with higher multi-index. A periodic homotopy path can be constructed relative to a common subgroup of 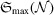 and 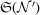, for example, by interpolating between coordinates, and this path is often crossing free and so a periodic isotopy.

Determination of maximal symmetry is a highly nontrivial task. The only general approach to the problem was proposed by Baburin (2016 ▸), based on subgroup relations between automorphism groups of connected components and a respective HRN. Along these lines maximal symmetry embeddings and their symmetry groups have been determined for *n*-grids as in Section 8.5.

### 7.3. Counting periodic isotopy classes by counting orbits   

Let us now consider embedded nets 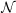 with *n* components on which the space group acts transitively. We are interested in calculating the number of periodic isotopy classes for a given topology. In the next section we solve this problem for *n*-fold **pcu** by reducing the counting to a combinatorial calculation, namely to a calculation of the number of orbits of a finite set of ‘normalized’ *n*-pcu nets under the action of a finite group of isometries, where the finite group is generated by cube rotations and shifts.

The method is generally applicable but for a translationally transitive *n*-pcu embedding a normalization of 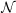 takes a particularly natural form in which the components have integral coordinates. While a normalized net is not uniquely associated with 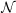 it turns out that their multiplicity corresponds to the cardinality of an orbit under the finite group action, and so counting the number of orbits gives the count we seek. A standard formula for counting such orbits is given by Burnside’s lemma which states the following. Let *G* be a finite group acting on a finite set *X* with group action *x* → *g* · *x*, so that the orbit of an element *z* ∈ *X* is the set {*g* · *z* : *g* ∈ *G*}. Then the number of distinct orbits is given by
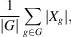
where *X*_*g*_ denotes the set of points *x* with *g* · *x* = *x*. In this way the problem is reduced to counting, for each symmetry element *g*, the number of normalized nets which have this symmetry.

## 8. Classifying multicomponent entangled nets   

We next determine the number of periodic isotopy types of various families of embedded nets (linear 3-periodic nets) in 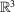 whose components are embeddings of the net **pcu**. The simplest family here consists of those nets 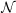 with *n* parallel components, each being a shifted copy of the model net 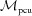. In this case we refer to 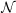 as a multigrid or *n*-grid. Such nets have dimension type {3; 3} and are shift-homogeneous.

For practical purposes, both in this section and in Section 9, we focus on the following hierarchy of four equivalence relations for embedded nets:

(1) Nets 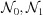 are affinely equivalent (respectively, orientedly affine equivalent) if they have translations 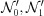 with 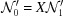 for some invertible 3 × 3 matrix *X* (respectively, with 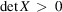).

(2) The pairs 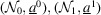, with given periodicity bases, are strictly periodically isotopic if there is a continuous path of embedded nets 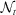 with an associated continuous path of periodicity bases from 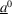 to 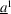.

(3) 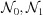 are periodically isotopic if they have strictly periodically isotopic pairs for some choice of periodicity bases 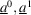.

(4) 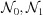 are topologically isomorphic, or have the same topology, if their structure graphs (underlying nets) are isomorphic as countable graphs.

### 8.1. Translation-transitive *n*-grids   

We first consider embeddings of *n*-grids with a strong form of homogeneity. Specifically we give group–supergroup methods which determine the periodic isotopy types of translation-transitive *n*-grids.

Considering the translation-transitivity assumption, it follows that the shift vectors relating parallel copies of a single-component grid are in fact coset representatives of some lattice with respect to the sublattice generated by the standard periodicity basis of a connected component. The number of cosets is equal to the index of a sublattice. This observation gives a recipe for generating translation-transitive *n*-grids, by enumerating superlattices of index *n* for the lattice of a connected component while discarding the associated *n*-grids which fail to be noncrossing.

A determination of index-*n* superlattices can be made with the following lemma [see also Cassels (1997 ▸), Davies *et al.* (1997 ▸)].

**Lemma 8.1**

Let *n* have a factorization *n* = *p*_1_ *p*_2_ *p*_3_, with 1 ≤ *p*_*i*_ ≤ *n*, and let
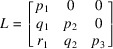
be a matrix with integral entries satisfying 0 ≤ *q*_1_ < *p*_2_, 0 ≤ *q*_2_ < *p*_3_ and 0 ≤ *r*_1_ < *p*_3_. The rows of the inverse matrix *L* ^−1^ generate a superlattice of 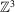 of index *n*. Moreover, every superlattice of 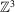 of index *n* has such a representation.

A computational determination of the number, β_tt_(*n*), of periodic isotopy types can now be implemented with the following three-step algorithm. Some of the values are recorded in the summary Table 1 ▸. (i) Using the lemma, generate all superlattices with index *n*. (ii) Discard such a superlattice if its corresponding *n*-grid has edge crossings. (iii) Reduce the resulting list to a (maximal) set of superlattices which are pairwise inequivalent under the point group of a primitive cubic lattice.

We have indicated that this (practical) three-step generation-and-reduction algorithm gives the number of congruence classes of translationally transitive *n*-grids. That this number also agrees with the (*a priori* smaller) number of periodic isotopy classes (up to chirality) is essentially a technical issue. This follows from Theorem 8.2[Statement theorem8.2] (iii) and Appendix *A* [App appa]. Moreover, for the same reason the algorithm determines exactly the translationally transitive *n*-grids which are maximal symmetry periodic isotopes.

We remark that a similar three-step algorithm can be applied in the case of translationally transitive embeddings of *e.g. n*-fold **dia**, *n*-fold **srs** and other nets. We conjecture that if connected components are crystallographic nets in their maximal symmetry configurations, then step (iii) leads directly to the classification into periodic isotopy classes.

### 8.2. A combinatorial enumeration of *n*-grids   

We now consider the wide class of general multigrids, with no further symmetry assumptions. The combinatorial objects relevant to periodic isotopy type counting are given in terms of various finite groups acting on finite sets of patterns which we now define.

Let *T* = {1, …, *n*}^3^, viewed as a discrete 3-torus, and let *C*_*n*_ be the cyclic group of order *n*. In particular *C*_*n*_ can act on *T* by cyclically permuting one of the three coordinates. Also, let *R* be the rotation symmetry group of the unit cube [0, 1]^3^. Then *R* acts on the discrete torus *T* in the natural way.

Let 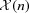 be the finite set of unordered *n*-tuples, or patterns, {*p*_1_, …, *p*_*n*_} where the points lie in *T* and have distinct coordinates, so that for all pairs *p*_*i*_, *p*_*j*_ the difference *p*_*i*_ − *p*_*j*_ has nonzero coordinates. In particular 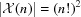. These *n*-tuples in fact correspond to the coordinates of the nodes appearing in a unit cell of the *n* components of a normalized *n*-grid.

Finally, for 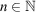, let ρ(*n*), α(*n*), β(*n*), respectively, be the number of orbits in 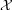 under the natural action of the groups
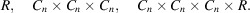
Recall that a linear graph knot for an embedded net 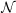 is determined by a choice of periodicity basis 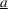 and is denoted 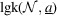. In the case of an *n*-grid 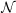 with its standard periodicity basis 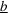 we refer to 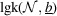 as the standard linear graph knot for 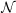. Evidently 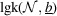 appears as the union of *n* disjoint translates in the flat 3-torus of *K*_pcu_.

**Theorem 8.2**

(i) The number of linear isotopy types of standard linear graph knots of *n*-grids is α(*n*). (ii) The number of rotational isotopy types of standard linear graph knots of *n*-grids is β(*n*). (iii) The number of periodic isotopy classes of *n*-grids is β(*n*).

The proof of this theorem is given in Appendix *A* [App appa]. The essential argument involves a discretization in which, in (ii) for example, the components are separately shifted by a (joint) isotopy to an evenly spaced position. Then *n* nodes in a unit cell correspond to a pattern of *n* coordinate distinct points in the discrete torus {1, 2, …, *n*}^3^. Additionally, for (iii) one must resolve the technical problem in Remark 11.1[Statement remark11.1] in the case of *n*-grids and show that the triple cyclic order of coordinates (modulo the rotation group *R*) is indeed a periodic isotopy invariant. We do this in Lemma 11.2[Statement lemma11.2], and the equivalence given in Proposition 6.5[Statement proposition6.5] is a helpful step in the proof. We also note that the periodic isotopy that one needs to construct in the proof, when the cyclic orders coincide modulo *R*, is simply a concatenation of a periodic isotopy of local component translations (to achieve equal spacing), followed by an elementary periodic isotopy induced by a path of affine motions corresponding to a (bulk) rotation and final translation.

### 8.3. Translational isotopy and framed *n*-grids   

The general formulation of periodic isotopy of necessity entails some technical complexity in the proofs. We now note two restricted but natural *n*-grid contexts where the determination of the number of equivalence classes simplifies. We omit the formal proofs. In the first of these we define a more restricted form of isotopy while in the second context we distinguish, or colour, one of the component grids.

Let us say that a multi-grid is aligned if its components 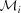 are translates of the model net 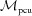 with node set 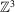.

**Definition 8.3**

Two aligned *n*-grids 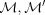 are translationally isotopic if for some labelling of the components there are continuous functions 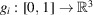, for 1 ≤ *i* ≤ *n*, with *g*_*i*_(0) = 0 for all *i*, such that

(i) for each *t* the embedded net
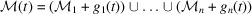
is a (noncrossing) linear 3-periodic net,

(ii) 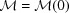 and 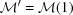.

This simple form of the periodic isotopy 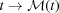 in fact corresponds to strict periodic isotopy for these nets with respect to the standard periodicity basis. It is a form of ‘local’ periodic isotopy in the sense that the deformation paths of the nodes are localized in space. In particular deformation paths incorporating bulk rotations are excluded.

**Theorem 8.4**

The number of translational isotopy classes of aligned *n*-grids is α(*n*).

For the second variation, let us define a framed *n*-grid to be an (*n* + 1)-grid with a distinguished component, the framing component. Thus a framed *n*-grid is a coloured *n* + 1 grid where all but one of the components are of the same colour. Periodic isotopy for coloured *n*-grids may be defined exactly as before but with the additional requirement that the maps (*f*_*t*_) respect colour.

It is evident that the cube rotation group *R* acts naturally on such framed *n*-grids. Also, as indicated in our remarks following Theorem 8.2[Statement theorem8.2], counting periodic isotopy types reduces to counting orbits of patterns *p* of *n* + 1 coordinate-disjoint points, *p* = (*p*_1_, …, *p*_*n*+1_), in the discrete torus {1, 2, …, *n* + 1}^3^. However, in view of the colour preservation we may assume, by shifting, that *p*_*n*+1_ lies in the *R*-orbit of (1, …, 1), and from this it follows (varying the proof of Theorem 8.2[Statement theorem8.2]) that the periodic isotopy classes correspond to the *R*-orbits of the *n*-tuples (*p*_1_, …, *p*_*n*_).

**Theorem 8.5**

The number of periodic isotopy classes of framed *n*-grids is ρ(*n*).

### 8.4. Employing Burnside’s lemma   

We can now make use of Burnside’s lemma to compute values of α(*n*), β(*n*) and ρ(*n*). The following formula readily shows that α(5) = 128, α(7) = 74088 for example.

**Proposition 8.6**

Let *p* be a prime number. Then
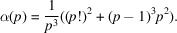

**Proof**

Note that a group element *g* = *abc* ≠ 000 in *C*_*p*_ × *C*_*p*_ × *C*_*p*_ with *a* or *b* or *c* equal to 0 does not fix any pattern under the cyclic action on *T* = {1, 2, …, *p*}^3^ and so 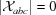 in this case. Also, every pattern is fixed by the identity element and so 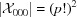. It remains to consider the (*p* − 1)^3^ group elements *abc* with none of *a*, *b*, *c* equal to 0.

The group element 111 acts as a diagonal shift and so any fixed pattern of nodes in *T* is determined by the unique node occupying a particular face of *T*. Conversely any of the *p* ^2^ node locations on this face determines a unique fixed pattern for the action of 111. Thus 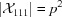.

Since *p* is prime the same argument applies to any group element *abc* with none of *a*, *b*, *c* equal to the identity element 0, since *a*, *b*, *c* each have order *p*. There are (*p* − 1)^3^ such elements *abc* and so the formula now follows from Burnside’s lemma.

□

The case of composite *n* is similar. In the case that each of *a*, *b*, *c* have order *r* where *r* divides *n* the size of the fixed set 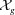 for *g* = *abc* is the product 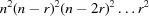. All other elements except the identity have no fixed patterns. In this way we obtain α(4) = 12 and α(6) = 2424.

Similarly, for the framed *n*-grids one may compute ρ(2) = 1, ρ(3) = 4, ρ(4) = 30. Evidently there is a rapid subsequent growth rate since the Burnside lemma formula quickly leads to the lower bound ρ(*n*) ≥ (*n*!)^2^/(24 × *n* ^3^).

### 8.5. Classes of embedded *n*-**pcu**   

**Figure 9:**
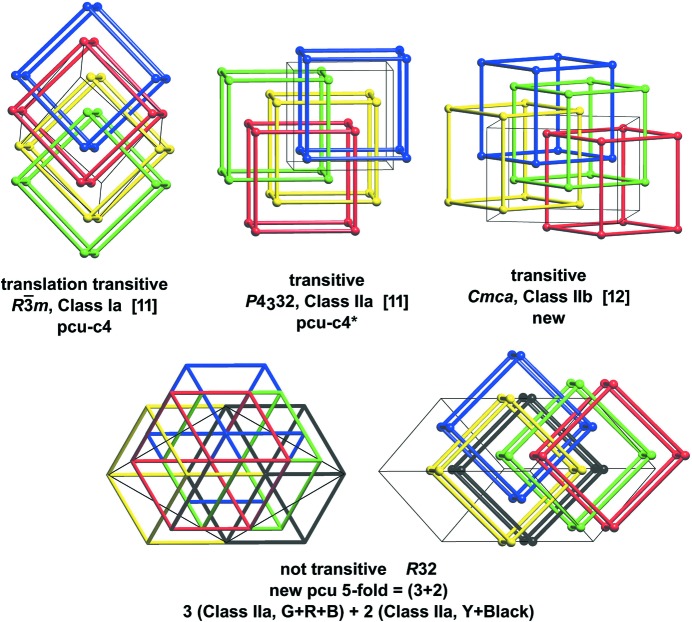
Four examples of shift-homogeneous *n*-grids with interpenetration class according to Baburin *et al.* (2005 ▸), the vertex and edge transitivity [*ve*] and the RCSR names where available.

Fig. 9 ▸ gives examples of small *n*-grids with contrasting transitivity properties. For more details, see the supporting information.

In Table 1 ▸ we summarize the number of classes of *n*-grids for various types of *n*-grid and forms of isotopy for some small values of *n* with the values of α(*n*) and β(*n*) obtained via Burnside’s lemma as before. The count β_*t*_(*n*) is for transitive *n*-grids in the sense given in Section 2.4, and for *n*-grids this coincides with vertex transitivity. The count β_tt_(*n*) is for translation-transitive *n*-grids which have components that are equivalent by translations in the space group. These counts, which coincide if *n* is prime, are obtained using the group–supergroup algorithm of Section 7.2.

**Figure 10:**
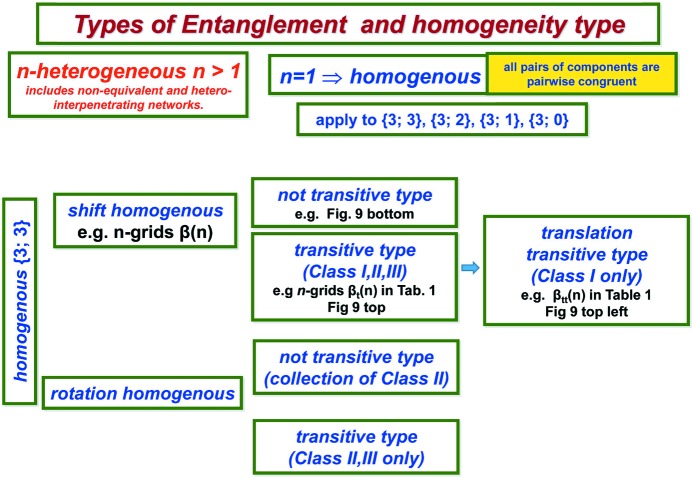
Homogeneity and transitivity types of entangled nets.

Fig. 10 ▸ summarizes homogeneity and transitivity types of multicomponent embedded nets.

## 9. Classifying lattice nets   

### 9.1. Depth-1 disconnected nets with a single-vertex QG   

A model net 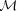 which has adjacency depth 1 with respect to the standard basis 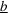 is determined by a set *F*_*e*_ of edge representatives [*a*, *b*] for the translational orbits of edges. In the case that there is a single orbit for the nodes we may assume that there is a node at the origin and choose the unique edge-orbit representative [*a*, *b*] such that (*a*, *b*) is a subset of the semi-open cube [0, 1)^3^. Such representative edges are determined up to sign by the vectors *a* − *b*, or equivalently in this case, by the labels of the depth-1 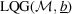. We use the following terminology for edges in *F*_*e*_. This will also be useful in subsequent sections.

The three axial edges are denoted *a*_*x*_, *a*_*y*_, *a*_*z*_ and *d*_1_, …, *d*_4_ denote the four diagonal edges which are incident to (0, 0, 0), (1, 0, 0), (1, 1, 0), (0, 1, 0), respectively. The three face diagonal edges which are incident to the origin are denoted *f*_*x*_, *f*_*y*_, *f*_*z*_, corresponding to the directions (0, 1, 1), (1, 0, 1), (1, 1, 0), while the edges *g*_*x*_, *g*_*y*_, *g*_*z*_ are the other face diagonals, parallel to the vectors (0, 1, −1), (1, 0, −1), (1, −1, 0), respectively. Thus we may define any set *F*_*e*_ by means of an ordered subword *w* of the ordered word
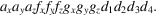
In view of the noncrossing condition it is elementary to see that every model net 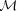 is affinely equivalent, simply by rotations, to a standardized model net defined by the standard ordered word of the form *w* = *w*_1_ *w*_2_ *d*_1_ or *w*_1_ *w*_2_, where *w*_1_ is either *a*_*x*_, *a*_*x*_ *a*_*y*_, *a*_*x*_ *a*_*y*_ *a*_*z*_ or the null word, and *w*_2_ is a face subword with zero, one, two or three letters, of which there are 27 possibilities.

We now determine the depth-1 embedded bouquet nets that are disconnected, that is, which have more than one and possibly infinitely many connected components. It turns out that there are six embedded nets up to affine equivalence and we now give six model nets for these types.

(i) 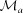 is the model net determined by *F*_*e*_ = {*a*_*x*_} and consists of parallel copies of a 1-periodic linear subnet.

(ii) 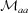 is determined by the word *a*_*x*_ *a*_*y*_ and is the union of parallel planar embeddings of **sql**.

(iii) 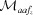 is the net for *a*_*x*_ *a*_*y*_ *f*_*z*_ and is the union of parallel planar embeddings of **hxl**.

(iv) 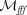 is the net for *f*_*x*_ *f*_*y*_ *f*_*z*_ and is the translation-transitive union of two disjoint copies of an embedding of **pcu**.

(v) 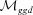 is the net for *g*_*x*_ *g*_*y*_ *d*_1_ and is the translation-transitive union of three disjoint copies of an embedding of **pcu**.

(vi) 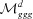 is the net for *g*_*x*_ *g*_*y*_ *g*_*z*_ *d*_1_ and is the translation-transitive union of three disjoint copies of an embedding of **hex**.

**Figure 11:**
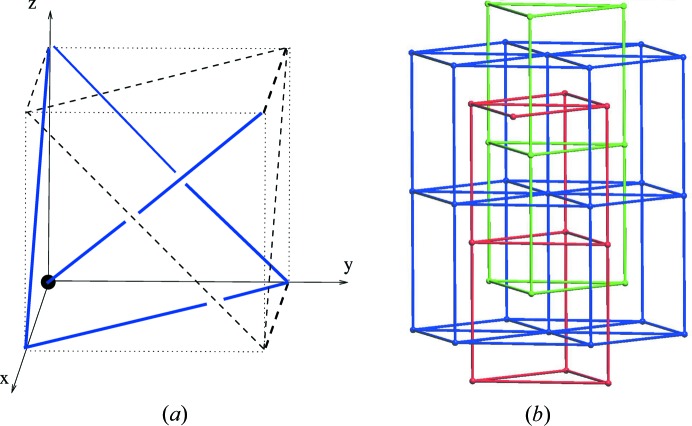
(*a*) A single-vertex quadruple-edged building block for the model net 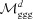. (*b*) A (rescaled) fragment of this net. The diagonal edge in the building block gives a 3^2^-penetrating edge.

In the above list, and in Tables 1 ▸, 3 ▸, we use a compact notation where the letter subscripts for the nondiagonal edges are suppressed if they appear in alphabetical order, and where *d* indicates the diagonal edge *d*_1_. Thus, the model net for *w* = *g*_*x*_ *g*_*y*_ *g*_*z*_ *d*_1_, which could be written as 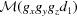, is written in the compact form 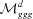. Its repeating unit, or motif, is indicated in Fig. 11 ▸ along with a fragment of the embedding rotated so that the penetrating edges are vertical.

**Theorem 9.1**

There are six affine equivalence classes of disconnected embedded nets with adjacency depth 1 and a single-vertex quotient graph.

**Proof**

Let 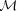 be a model net of the type stated, with generating edge set *F*_*e*_ with |*F*_*e*_| = *m*. If *m* = 1 (respectively, *m* = 2) then 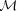 is affinely equivalent to 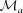 (respectively, 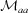).

Let *m* = 3. Then the three edges of *F*_*e*_ have separate translates, under the periodic structure, to three edges in 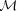 which are incident to a common node. Suppose first that this triple is coplanar. Then it determines a planar subnet, 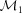 say, which is an embedding of **hxl**. Also 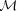 is equal to the union of the translates of 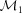 of the form 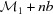 where *b* is a vector of integers and 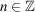. Thus 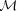 is affinely equivalent to 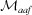.

On the other hand, if the edges of *F*_*e*_ are not coplanar then 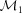 is an embedding of **pcu**. Examination shows that this occurs with 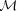 disconnected, only for words *w* of the forms (i) *fff*, giving two components, (ii) *fgg*, *gfg* or *ggf*, each of which is of type *fff* after a translation and rotation, and (iii) *ggd*, which gives three components.

For *m* ≥ 4 the model net 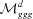 is the only net which is not connnected.

□

### 9.2. Connected lattice nets with depth 1   

Let 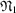 denote the family of proper linear 3-periodic nets with a periodic structure basis providing a depth-1 LQG with a single vertex. We now consider the subfamily 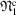 of connected nets 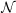 in 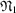. These nets also give building block nets for embedded nets with a double-vertex quotient graph, and for multicomponent nets. In Theorem 9.5[Statement theorem9.5] and Corollary 9.6[Statement corollary9.6] we classify the nets up to oriented affine isomorphism and up to periodic isotopy, respectively, there being 19 classes for each equivalence relation. As in the previous section it will suffice to consider model nets. Moreover each model net 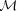 in 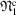 is determined by an ordered word for the edges of a repeating unit *F*_*e*_ and these edges [*a*, *b*] are subsets of the unit cell [0, 1)^3^ except for one of their endpoints. In view of connectivity and noncrossing conditions the 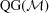 is a bouquet graph with a single vertex and loop edges of multiplicity *m* = 3, 4, 5, 6 or 7 (as implied by Lemma 4.4[Statement lemma4.4]).

To distinguish these model nets we make use of some new readily computable local features which can be read off from the repeating unit and which provide some readily computable structural invariants under affine isomorphism.

**Definition 9.2**

The hxl-multiplicity 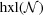 of an embedded net 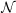 is the number of translation classes of planar 2-periodic subnets of 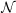 which are completely triangulated.

For the model nets 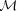 in 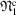 this multiplicity is equal to the number of triples of edges [*a*, *b*] in *F*_*e*_ whose edge vectors, *b* − *a*, form a coplanar triple. It may also be computed from the PS as the number of 3-cycles divided by 6. Thus the PS of **fcu** is 3^24^.4^36^.5^6^ and so 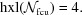


The next definition might be viewed as a strong form of local catenation.

**Definition 9.3**

(i) An edge of an embedded net is 3^2^-penetrating if there exist two disjoint parallel edge-cycles of length 3 and an edge [*a*, *b*] which passes through them in the sense that the open line segment (*a*, *b*) intersects the convex hull of each cycle. (ii) An edge of an embedded net is 4^2^-penetrating if it passes through two disjoint parallel untriangulated parallelograms.

**Figure 12:**
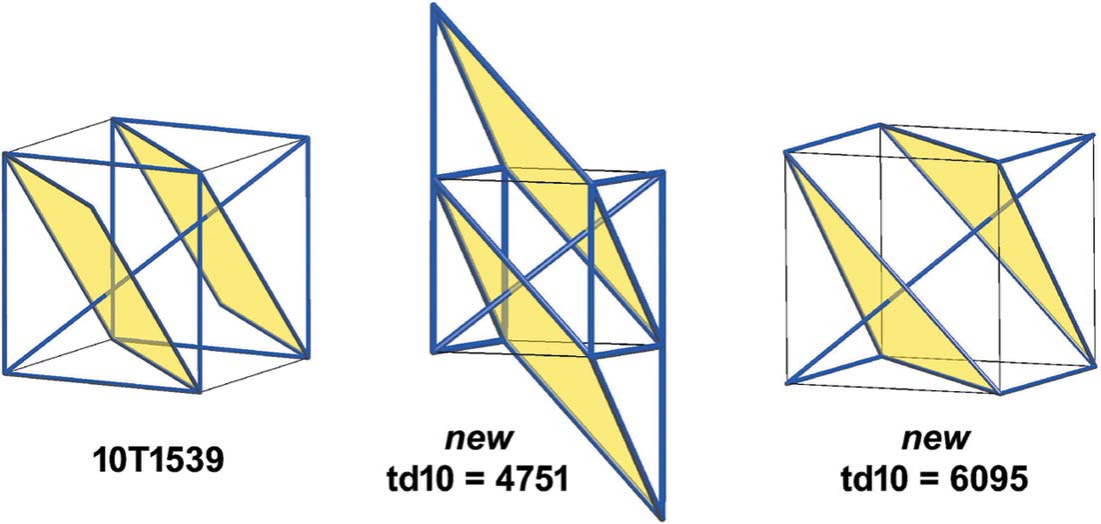
Penetrating edges of type 3^2^ and 4^2^ as observed for three 10-coordinated nets from Table 3 ▸.

One can check for example that for the model net 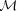 in 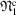 with a defining ordered word *w* there exists a 3^2^-penetrating edge if and only if *w* contains the subword *g*_*x*_ *g*_*y*_ *g*_*z*_ *d*_1_. Also there exists a 4^2^-penetrating edge if and only if *w* contains *d*_1_ and precisely two of the three letters *g*_*x*_, *g*_*y*_, *g*_*z*_. See Fig. 12 ▸.

We similarly define when an edge is 3^1^-penetrating or 4^1^-penetrating. In fact there are no depth-1 lattice nets with a 3^1^-penetrating edge. In general let us say that 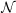 has property 3^*k*^ if there are 3^*k*^-penetrating edges but no 3^*k*+1^-penetrating edges. We also define property 4^*k*^ similarly. We indicate these properties in column 5 of Table 3 ▸.

### 9.3. Classification of depth-1 lattice nets   

**Figure 13:**
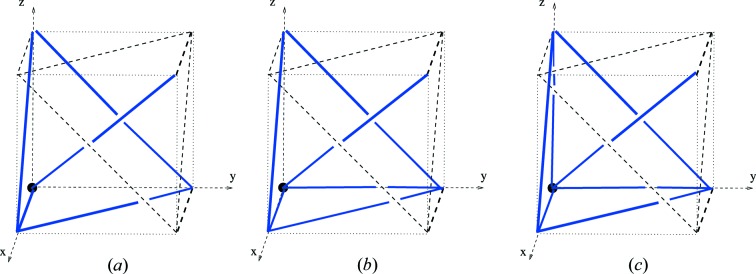
Motifs for the model nets (*a*) 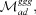 (*b*) 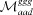 and (*c*) 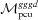 (14T957) which have a 3^2^-penetrating edge.

We now define 19 model nets 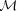 in 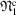 with standard orthonormal basis as a depth-1 periodicity basis and where in each case the node set is the subset 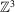 of 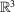. We do this, as in the previous section, by specifying a defining edge word, as listed in column 2 of Table 3 ▸. The nine nets without the strong edge penetration property (of type 3^2^ or 4^2^) appear in the RCSR whereas the other ten nets do not. This reflects the fact that the strongly penetrated nets can be viewed as exotic forms in reticular chemistry. Indeed, there are three new topologies which have not been observed either in the RCSR or the *ToposPro* net databases. Two of these are provided by the model nets 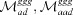 given in Fig. 13 ▸.

We also record in the final column the cardinality of the point group of the maximal symmetry net with the given topology, which we may denote by π(**pcu**) *etc*.

Let us define an elementary affine transformation of 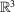 to be a rotation, a translation or a linear map whose representing matrix has entries 1 on the main diagonal and a single nonzero nondiagonal entry equal to 1 or −1. These maps, such as (*x*, *y*, *z*) → (*x* − *z*, *y*, *z*), map model nets to model nets and play a useful role in case-by-case analysis.

**Remark 9.4**

We note that the countable graph **ilc**, represented by the model net 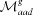, can be represented in other ways. The model net 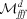 gives one such alternative. The topology is also made apparent by its equivalence, by elementary transformations, with the net obtained from the **pcu** model net by the addition of integer translates of the long diagonal edges with edge vector (1, 2, 1). However, in this case the standard basis is a periodic structure basis of depth 2.

**Theorem 9.5**

There are 19 oriented affine equivalence classes of connected lattice nets with depth 1.

We have obtained this classification by means of a case-by-case proof as well as a verification by an enumeration of lattice nets using *GAP*. The following interesting special case, with two new nets, illustrates the general proof method. (See the supporting information for the complete proof.)

**Determination of the 10-coordinated connected lattice nets of depth 1**

Suppose first that a model net 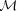 in this case has three axial edges and two face edges. Then it is straightforward to see that it is equivalent by elementary affine transformations to the model net 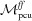, for **bct**. Also, any model net of type *aaafd* is similarly equivalent to this type. On the other hand, a type *aaagd* model net has 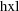-multiplicity equal to 1, rather than 2, and so represents a new equivalence class. Its topology is **ile**.

Consider next the model nets with two axial edges and no diagonal edges. These are equivalent by elementary affine transformations to a model net with three axial edges and so they are equivalent to the model nets in Table 3 ▸ for **bct** and **ile**. The same is true for the nine nets of type *aawd* where *w* is a word in two facial edges which is not of type *gg*.

Thus, in the case of two axial edges it remains to consider the types *a*_*x*_ *a*_*y*_ *wd*_1_ with *w* = *g*_*x*_ *g*_*y*_, *g*_*x*_ *g*_*z*_ and *g*_*y*_ *g*_*z*_. Each of these has a penetrating edge of type 4^2^. The first two are model nets in the list and they give new and distinct affine equivalence classes in view of their penetration type and differing 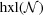 count. The third net, for the word *a*_*x*_ *a*_*y*_ *g*_*y*_ *g*_*z*_ *d*_1_, is a mirror image of the first net and is orientedly affinely equivalent to it, by Remark 5.5[Statement remark5.5] for example.

It remains to consider the case of one axial edge, *a*_*x*_, together with *d*_1_ and three facial edges. If there are two edges of type *f*_*x*_, *f*_*y*_ or *f*_*z*_ then there is an elementary equivalence with a model net with two axial edges. The same applies if there is a single such edge. [For an explicit example consider *a*_*x*_ *f*_*x*_ *g*_*y*_ *g*_*z*_ *d*_1_ and check that the image of this net under the transformation (*x*, *y*, *z*) → (*x*, *y* − *z*, *z*) gives a depth-1 net with two axial edges.]

Finally the model net for *a*_*x*_ *g*_*x*_ *g*_*y*_ *g*_*z*_ *d*_1_ appears in the listing and gives a new class with penetration type 3^2^.

□

**Corollary 9.6**

There are 19 periodic isotopy classes of connected linear 3-periodic nets in 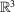 with adjacency depth 1 and a single-vertex quotient graph.

**Proof**

If the connected linear 3-periodic nets 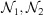 are orientedly affinely equivalent then, as previously observed, they are periodically isotopic. Thus there are at most 19 periodic isotopy classes. On the other hand, periodically isotopic embedded nets have structure graphs which are isomorphic as countable graphs. Since the 19 model nets have nonisomorphic structure graphs the proof is complete.

□

**Figure 14:**
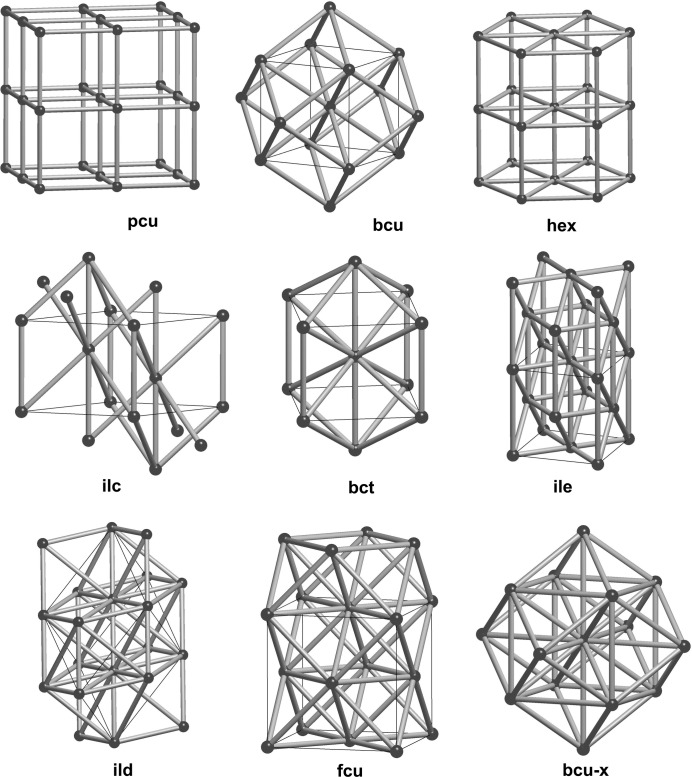
Maximal symmetry embeddings of the nine model nets of 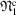 which do not have the 3^2^- or 4^2^-penetration property.

Theorem 9.5[Statement theorem9.5], together with the linear implementation of graph isomorphisms indicated in Theorem 4.8[Statement theorem4.8], implies that the structure graphs of the 19 model nets must be nonisomorphic as graphs. This also follows on examining the td10 topological density count. We remark that Table 3 ▸, without the final td10 column, almost distinguishes 19 affine equivalence classes (and hence, by Theorem 4.8[Statement theorem4.8], the structure graphs) since we have only appealed to topology density to distinguish the curious pair 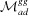 (8T17), 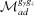 (8T21). Fig. 14 ▸ shows the maximal symmetry embeddings of the nine model nets of 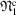 which do not have the 3^2^- or 4^2^-penetration property.

## 10. Double lattice nets and further directions   

We give a brief indication of research directions in the determination of periodic isotopy classes and periodic isotopes for embedded nets with a double-vertex quotient graph as well as research directions in rigidity and flexibility.

### 10.1. Double lattice nets   

For convenience we define a double lattice net to be an embedded periodic net 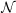 in 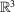 whose set of nodes is the union of two translationally equivalent rank-3 lattices and we let 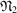 be the family of proper double lattice nets with adjacency depth 1.

The double-vertex quotient graph 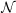 in 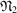 consists of two bouquet graphs and a number of nonloop edges. We denote these graphs as *H*(*m*_1_, *m*_2_, *m*_3_) where *m*_1_ and *m*_3_ are the loop multiplicities, with *m*_1_ ≥ *m*_3_ ≥ 0, and *m*_2_ is the multiplicity of the connecting edges. From Lemma 4.4[Statement lemma4.4] we have the necessary conditions 0 ≤ *m*_1_ ≤ 7 and 0 ≤ *m*_2_ ≤ 8 as well as *m*_3_ ≥ 1 if *m*_2_ = 1, since, from the definition of a linear 3-periodic net, there can be no nodes of degree 1. If 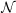 is a net in 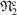, the subfamily of connected nets, then we also have the additional condition *m*_2_ ≥ 1.

Each net 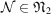 admits a unique threefold decomposition 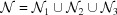 where 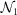 and 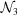 are the disjoint 3-periodic subnets associated with the two bouquet subgraphs and where 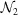 is the net, with the same node set as 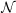, associated with the subgraph with nonloop edges. The subnets 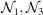 may have no edges if one or both vertices has no loop edges. When loops are present on both vertices then the nets 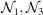 are bouquet nets, and are of three possible dimension types, namely {3; 1}, {3; 2} or {3; 3}. As we have seen earlier, for type {3; 1} there is one affine isomorphism class of embedded nets, for type {3; 2} there are two such classes and for type {3; 3} there are three classes for disconnected nets and 19 classes for connected nets.

Thus in the threefold decomposition of a net 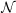 in 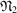, each of the subnets 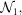 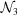 is either devoid of edges or is separately orientedly affinely equivalent to one of the 25 model nets for 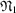. The relative position (parallel or inclined, for example) of these component nets allows for considerable diversity for the entangled net 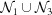. In particular, while 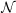 is affinely equivalent to a general model net 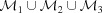, with standard periodic structure basis 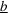, in general we can only additionally arrange that one of the subnets 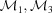 is equal to a translate of one of the specific 25 model nets in Tables 2 ▸ and 3 ▸.

Evidently there is a considerably diversity for the periodic isotopy classes of embedded nets with depth 1 and a double-vertex quotient graph. We now show that there is even a marked increase in the number of topologies for such nets.

For 1 ≤ *m* ≤ 8 define 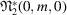 to be the family of nets 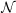 in 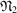 which have a periodicity basis with a depth-1 bipartite quotient graph *H*(0, *m*, 0) with an edge carrying the label (0, 0, 0). The label condition here ensures the natural condition that 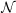 has an edge between the pair of representative joints in the semi-open unit cell for the periodicity basis. In fact this convention, which we call the unit-cell property, is the natural convention used by Chung *et al.* (1984 ▸) in their schemes for the enumeration of periodic nets.

For *m* = 1, 2, 3 the nets of this type are not connected. For *m* = 4 it is well known that there is a unique connected topology 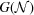 for the nets in 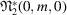, namely the diamond net **dia** (Beukemann & Klee, 1992 ▸). For higher values of *m* we are able to determine the topologies through a computational analysis based in part on the indivisibility criterion Proposition 4.5[Statement proposition4.5]. See also the supporting information.

**Proposition 10.1**

There are 117 nonisomorphic topologies for bipartite double lattice nets with the unit-cell property, which are connected and have adjacency depth 1. Moreover, the numbers of *m*-coordinated topologies, for *m* = 4, 5, 6, 7 and 8, are, respectively, 1, 11, 31, 40 and 34.

### 10.2. Rigidity and flexibility   

The analysis of infinitesimal rigidity and flexibility for connected crystal frameworks 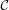 is a well-developed mathematical topic. In its simplest form a velocity field on the node set is assumed to be periodic with respect to a given periodicity basis 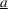. This is the so-called fixed lattice theory and in fact it corresponds exactly to the rigidity theory of fixed edge-length graph knots on a fixed flat torus for the parallelepiped defined by the periodicity basis. In this case a finite matrix, the periodic rigidity matrix for the pair 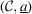, determines the space of periodic infinitesimal flexes and so this matrix is a discriminator for the (strict) periodic rigidity of 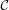 with respect to 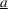. On the other hand, the flexible lattice theory allows for infinitesimal motions of the periodicity basis and so embraces a larger finite dimensional vector space of velocity fields with a correspondingly larger rigidity matrix (see Borcea & Streinu, 2010 ▸; Power, 2014*b* ▸). Recently, necessary and sufficient conditions have been given for infinitesimal rigidity with respect to the infinite dimensional space of all velocity fields (see Kastis & Power, 2019 ▸).

The fixed lattice theory also has close connections with the analysis of rigid unit modes (RUMs) in material crystals with a connected bond-node net. See for example the RUM mode analysis in the work of Badri *et al.* (2014 ▸), Power (2014*a* ▸). In fact this analysis also applies to disconnected crystal frameworks with several components if there are no interaction constraints between the components. Indeed, suppose that 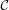 belongs to the interpenetration class and let 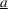 be a periodicity basis for both 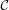 and each of its finitely many components 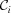. Then the RUM spectrum 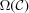 of 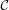, with respect to 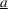, is the union of the RUM spectra of its components.

A crystal framework is said to be critically coordinated, or to be a Maxwell framework, if the quotient graph satisfies |*E*| = 3|*V*|. This is often interpreted as an equality between the total number of constraints (provided by |*E*| equations) that restrict the total number of degrees of freedom of a repeating unit of nodes, which is 3|*V*|. It also implies an equality of limits of averages over increasing volumes for these constraint/freedom quantities. It is for such frameworks, which includes all zeolite frameworks for example, that the RUM spectrum is typically a nontrivial algebraic variety exhibiting detailed structure (Dove *et al.*, 2007 ▸; Power, 2014*a* ▸; Wegner, 2007 ▸).

In the light of this it is of interest to determine the basic Maxwell frameworks 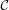 which have a depth-1 LQG with either one or two vertices. From Proposition 10.1[Statement proposition10.1] it follows that there are 31 topologies for crystal frameworks of this type with the unit-cell property and quotient graph *H*(0, 6, 0). These remarks suggest that it would be worthwhile to augment periodic net database resources with tools for the identification of Maxwell lattices and the calculation of flexibility information related to RUM spectra.

## Supplementary Material

Proof of Theorem 9.5. DOI: 10.1107/S2053273320000625/ib5087sup1.pdf

Coordinates for nets from Table 3 (in cgd format). DOI: 10.1107/S2053273320000625/ib5087sup2.txt

Coordinates for nets from Section 10 (in cgd format). DOI: 10.1107/S2053273320000625/ib5087sup3.txt

Coordinates for n-grids (corresponding to beta values) from Table 1 (in cgd format). DOI: 10.1107/S2053273320000625/ib5087sup4.txt

## Figures and Tables

**Figure 15:**
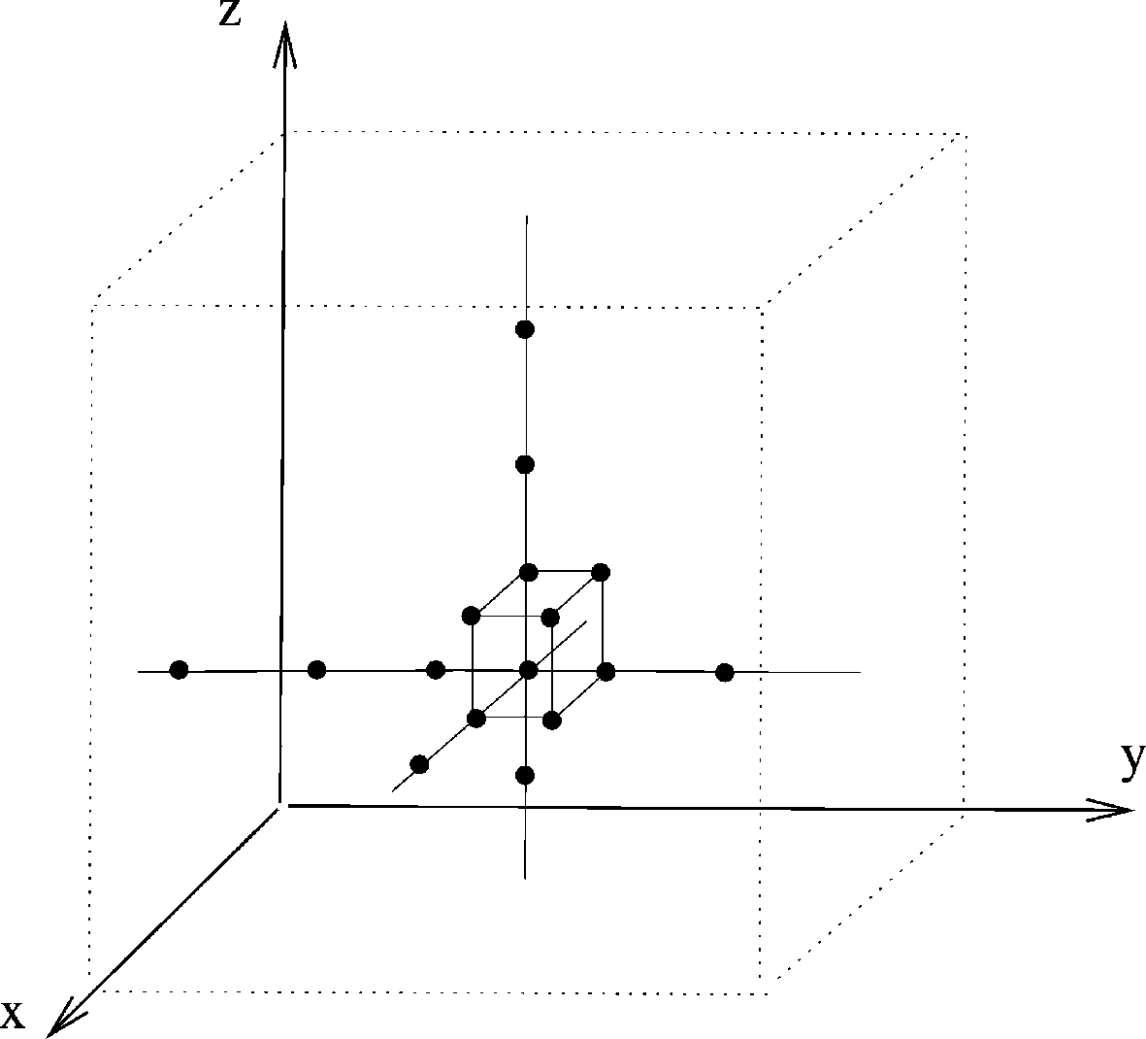
A chain subgraph knot of *k* · *K*_pcu_.

**Table 1:** Counts of isotopy classes of *n*-grids

*n*	2	3	4	5	6	7	*n*-grids/isotopy
α(*n*)	1	4	12	128	2424	74088	*n*-grids/translational isotopy
β(*n*)	1	1	3	9	89		*n*-grids/periodic isotopy
β_*t*_(*n*)	1	1	3	2	7	4	transitive *n*-grids/periodic isotopy
β_tt_(*n*)	1	1	1	2	1	4	translation-transitive *n*-grids/periodic isotopy

**Table 2:** Disconnected nets with a single-vertex, depth-1, labelled quotient graph

Model net	Edge word	Coordination	Net/multiplicity
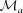	*a*_*x*_	2	line ∞
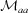	*a*_*x*_ *a*_*y*_	4	**sql** ∞
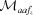	*a*_*x*_ *a*_*y*_ *f*_*z*_	6	**hxl** ∞
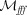	*f*_*x*_ *f*_*y*_ *f*_*z*_	6	**pcu-c**
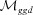	*g*_*x*_ *g*_*y*_ *d*_1_	6	**pcu-c3**
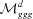	*g*_*x*_ *g*_*y*_ *g*_*z*_ *d*_1_	8	**hex-c3**

**Table 3:** Connected nets with a single-vertex, depth-1, labelled quotient graph

Model net	Edge word	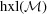	Coordination	Penetration	Topology	td10	π(-)
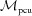	*a*_*x*_ *a*_*y*_ *a*_*z*_	0	6	0	**pcu**	1561	48
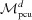	*a*_*x*_ *a*_*y*_ *a*_*z*_ *d*_1_	0	8	0	**bcu**	2331	48
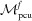	*a*_*x*_ *a*_*y*_ *a*_*z*_ *f*_*x*_	1	8	0	**hex**	2331	24
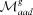	*a*_*x*_ *a*_*y*_ *g*_*x*_ *d*_1_	0	8	4^1^	**ilc**	3321	12
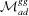	*a*_*x*_ *g*_*x*_ *g*_*y*_ *d*_1_	0	8	4^2^	8T17	4497	4
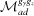	*a*_*x*_ *g*_*y*_ *g*_*z*_ *d*_1_	0	8	4^2^	8T21	4041	12
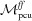	*a*_*x*_ *a*_*y*_ *a*_*z*_ *f*_*x*_ *f*_*y*_	2	10	0	**bct**	3101	16
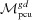	*a*_*x*_ *a*_*y*_ *a*_*z*_ *g*_*x*_ *d*_1_	1	10	4^1^	**ile**	3761	8
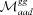	*a*_*x*_ *a*_*y*_ *g*_*x*_ *g*_*y*_ *d*_1_	0	10	4^2^	10T1539	4991	4
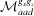	*a*_*x*_ *a*_*y*_ *g*_*x*_ *g*_*z*_ *d*_1_	1	10	4^2^	new	4751	4
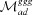	*a*_*x*_ *g*_*x*_ *g*_*y*_ *g*_*z*_ *d*_1_	1	10	3^2^	new	6095	4
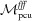	*a*_*x*_ *a*_*y*_ *a*_*z*_ *f*_*x*_ *f*_*y*_ *f*_*z*_	3	12	4^1^	**ild**	4201	12
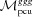	*a*_*x*_ *a*_*y*_ *a*_*z*_ *g*_*x*_ *g*_*y*_ *g*_*z*_	4	12	0	**fcu**	3871	48
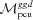	*a*_*x*_ *a*_*y*_ *a*_*z*_ *g*_*x*_ *g*_*y*_ *d*_1_	2	12	4^2^	12T1305	5191	4
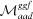	*a*_*x*_ *a*_*y*_ *g*_*x*_ *g*_*y*_ *f*_*z*_ *d*_1_	1	12	4^2^	12T1657	5431	12
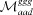	*a*_*x*_ *a*_*y*_ *g*_*x*_ *g*_*y*_ *g*_*z*_ *d*_1_	2	12	3^2^, 4^1^	new	6421	4
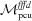	*a*_*x*_ *a*_*y*_ *a*_*z*_ *f*_*x*_ *f*_*y*_ *f*_*z*_ *d*_1_	6	14	4^1^	**bcu-x**	4641	48
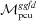	*a*_*x*_ *a*_*y*_ *a*_*z*_ *g*_*x*_ *g*_*y*_ *f*_*z*_ *d*_1_	4	14	4^2^	14T199	5631	12
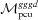	*a*_*x*_ *a*_*y*_ *a*_*z*_ *g*_*x*_ *g*_*y*_ *g*_*z*_ *d*_1_	4	14	3^2^, 4^1^	14T957	6621	12

## Appendix A Proof of Theorem 8.2[Statement theorem8.2]

Note first that any connected component *K*_*i*_ of *K* is determined by the position of its unique node in [0, 1)^3^. Thus *K* is determined by the position *p*_*i*_ = (*x*_*i*_, *y*_*i*_, *z*_*i*_), 1 ≤ *i* ≤ *n*, of its *n*-tuple of nodes. Also, in view of the disjointness of components two such nodes *p*_*i*_, *p*_*j*_ have differing corresponding coordinates in [0, 1). Consider a deformation path (*f*_*t*_) from *K* to *K*′. Since the graph knots *f*_*t*_(*K*) are also graph knots of *n*-grids, and edge collisions cannot occur in the deformation, it follows that the cyclical order of the *x*, *y* and *z* coordinates of the points *f*_*t*_(*p*_1_), …, *f*_*t*_(*p*_*n*_), is constant. Thus the ordered triple of cyclic orders for the coordinates is an invariant for linear graph knot isotopy.

Despite the constraint of coordinate distinctness we see that *K* can be linearly isotopic to an *n*-grid graph knot *K*′ determined by *p*_*i*_′ = (*x*_*i*_′, *y*_*i*_′, *z*_*i*_′), 1 ≤ *i* ≤ *n*, where the *n* coordinates *x*_*i*_′ lie at the midpoints of the distinct subintervals of the form [*j*/*n*, (*j* + 1)/*n*), 0 ≤ *j* ≤ *n* − 1. This spacing is achieved by simultaneously translating the points *p*_*i*_ in the *x* direction at appropriate independent speeds while maintaining *x*-coordinate distinctness. Additionally, the equal spacing of the *y* and *z* coordinates can be achieved by similar isotopies which locally translate in the *y* and *z* directions. The resulting position is unique up to the cyclic permutation action of *C*_*n*_ × *C*_*n*_ × *C*_*n*_ on the coordinate axes. It follows now that two graph knots of *n*-grids are linearly isotopic if the cyclic order of their coordinates coincides. Thus the set of cyclic orders is a complete invariant for linear graph knot isotopy and (i) and (ii) follow.

Assume next that the *n*-grids  and  are periodically isotopic. It will suffice to show that their linear graph knots are rotationally linearly isotopic.

Without loss of generality we may assume that the components have node sets that lie on translates of the lattice  in . Thus, by the definition of periodic isotopy there are periodicity bases  and , with integer entries, such that  and  are strictly periodically isotopic by means of a deformation path (*f*_*t*_) and an associated path of bases  from  to . Define *k*_1_ to be a common multiple of the *x* coordinates of {*a*_1_, *a*_2_, *a*_3_} and similarly define *k*_2_, *k*_3_ for the *y* and *z* coordinates. Then there is an implied periodic isotopy between  and , for some periodicity basis  with integer entries. This is given by the same periodic isotopy deformation path (*f*_*t*_) but with a new associated path of bases (for lower translational symmetry) which is determined by the initial basis  and the path . So, without loss of generality we may assume at the outset that .

We next show that  is equal to  where *k*′ is a cyclic permutation of *k*. Thus we will obtain that the linear graph knots   are rotationally linearly isotopic.

To see this consider a single component  of the *n*-grid . Note that the linear graph knot  has minimal discrete length cycles *c*_1_, *c*_2_, *c*_3_ with homology classes δ_1_, δ_2_, δ_3_, respectively, equal to the standard generators of the homology group  of the containing flat 3-torus. These discrete lengths are *k*_1_, *k*_2_, *k*_3_. Moreover we see, from the rectangular geometry of , the following uniqueness property, that if *c*_1_ and  are two such minimal length cycles for δ_1_ which share a node then *c*_1_ = . Indeed, the minimality implies that the edges of *c*_1_ are parallel or, equivalently, that the nodes of *c*_1_ can only differ in the *x* coordinate.

Let  be the corresponding component of . In fact . The linear graph knot  is, by definition, equal to the affine rescaling of the intersection of the body  with the semi-open parallelepiped defined by the periodicity vectors . We note that if  is not of the form  then for at least one of the standard generators δ_*i*_ (associated with *a*_*i*_′) of the flat 3-torus homology group , the minimal length cycles do not have the uniqueness property. This follows from elementary geometry since not all edges of the cycle can be parallel when  is not parallel to a coordinate axis.

On the other hand, the linear isotopy between  and  preserves the lengths of cycles of edges and so the claim follows. Since we have shown that  and  are isotopic linear graph knots up to a rotation, it follows from the technical lemma, Lemma 11.2[Statement lemma11.2], that the graph knots  and  are linearly isotopic up to a rotation, and so (iii) now follows from (ii).

□

Remark 11.1   Recall the notation  introduced in the proof of Theorem 6.2[Statement theorem6.2]. Let us say that this denotes the amplification by  of the periodic structure basis . We pose the following general problem. If  are linear periodic nets which have a common periodic structure basis  and if the pair  is periodically isotopic to the pair , then does it follow that  and  are periodically isotopic? In view of the proposition above this is equivalent to the corresponding problem for linear graph knots *K* and their *k*-fold amplifications which we may write as *k* · *K*. We expect that this is true and therefore that the amplified knots are isotopic if and only if the unamplified knots are isotopic. In fact one can verify this connection for various specific classes of interest, as we do below in the case of multigrid nets.

The following technical lemma resolves the question of Remark 11.1[Statement remark11.1] in the case of *n*-grids.

Lemma 11.2   Let  and  be shift-homogeneous *n*-grids with standard linear graph knots *K*_0_ and *K*_1_ and suppose that for some  the amplified graph knots *k* · *K*_0_ and *k* · *K*_1_ are isotopic. Then *K*_0_ and *K*_1_ are strictly linearly isotopic.

Proof   Fig. 15 ▸ indicates a subgraph knot, *C*_0_ say, of one of the components of *k* · *K*_0_ in the case that *k* = (3, 6, 5). We refer to this as a ‘chain’. It consists of a small cube of edges attached to three cycles of edges in the axial directions. Let *p*_1_ denote the vertex which is common to these three cycles. The other *n* − 1 components of *k* · *K*_0_ have similar chains which are shifts of *C*_0_ and there is a unique such chain where the shift of *p*_1_ lies in the semi-open small cube *p*_1_ + [0, 1)^3^ of the flat 3-torus. Let *p*_2_, …, *p*_*n*_ be these axial joints and let *J*_0_ = *J*_0_(*p*_1_, …, *p*_*n*_) be the union of these chains (giving a linear subgraph knot of *k* · *K*_0_). Suppose that *k* · *K*_0_ and *k* · *K*_1_ are linearly isotopic, by the isotopy (*g*_*t*_), 0 ≤ *t* ≤ 1. This restricts to a linear isotopy from *J*_0_ to a subgraph knot *g*_1_(*J*_0_) of *k* · *K*_1_. In this isotopy the images under *g*_*t*_, for 0 < *t* < 1, of the *n* axial cycles of *J*_0_ in a specific coordinate direction need not be linear. However, since there can be no collisions the cyclical order for *t* = 0 agrees with the cyclical orders for *t* = 1. It follows that *g*_1_(*J*_0_), which has the form *J*_0_(*q*_1_, …, *q*_*n*_), is a subgraph knot of *k* · *K*_1_ of the same cyclical type as the subgraph knot *J*_0_. Since *K*_0_ and *K*_1_ are also defined by the cyclical order of *p*_1_, …*p*_*n*_ and *q*_1_, …, *q*_*n*_ it follows that they are linearly isotopic. □
