# Supplementary material for: Isotopy classes for 3-periodic net embeddings
Source: Acta Crystallogr A Found Adv. 2020 Mar 5;76(Pt 3):275–301. doi: 10.1107/S2053273320000625 (PMC7233014; doi:10.1107/S2053273320000625)
Supplement: Supplementary file 1 [file a-76-00275-sup1.pdf]

FOUNDATIONS
ADVANCES

Volume 76 (2020)

Supporting information for article:

Isotopy classes for 3-periodic net embeddings

Stephen Power, Igor Baburin and Davide Proserpio

ISOTOPY CLASSES FOR 3-PERIODIC NET EMBEDDINGS

I. A. BABURIN, S. C. POWER AND D. M. PROSERPIO

Supporting Information

The proof of Theorem 9.5.

Proof. Let $\mathcal{M}$ be model net with adjacency depth 1 and a single vertex quotient graph. We show that $\mathcal{M}$ is equivalent to one of the 19 model nets by an elementary affine transformation.

The case $m = 3$**.** In all cases it is clear that $\mathcal{M}$ is equivalent to $\mathcal{M}_{\text{pcu}}$.

The case $m = 4$**.** We consider 4 subcases:

(i) Assume that 3 of the edges of F_e are axial edges. Then $\mathcal{M}$ is obtained from $\mathcal{M}_{\text{pcu}}$ by the addition of an additional edge to the motif. If this is a facial edge then, by rotation and translation $\mathcal{M}$ is equivalent to $\mathcal{M}^f_{\text{pcu}}$, the model net for the word $a_x a_y a_z f_x$. If the extra edge is a diagonal edge then $\mathcal{M}$ is equivalent to $\mathcal{M}^d_{\text{pcu}}$.

(ii) Assume that exactly 2 of the 4 edges of F_e are axial edges. We may these are a_x, a_y and we may also assume that neither of the remaining 2 edges is in the xy-plane since in this case there would be a triple of coplanar edges in F_e and $\mathcal{M}$ would be equivalent to $\mathcal{M}^f_{\text{pcu}}$. Suppose first that there is no diagonal edge and so $\mathcal{M}$ is of type $a_x a_y w$ with w one of $f_x f_y, f_x g_y, g_x f_y, g_x g_y$. These nets are pairwise equivalent by rotation about the z-axis and translation. By an elementary affine transformation they are thus all equivalent to $\mathcal{M}^d_{\text{pcu}}$.

Assume on the other hand that only 1 of the 2 extra edges is a facial edge. Translating and rotating we may assume that this edge is f_x. Also we may assume a noncoplanarity position of the diagonal edge with respect to f_x and a_x, as in Figure 1, since otherwise there is an oriented affine equivalence with the model net for **hex**.

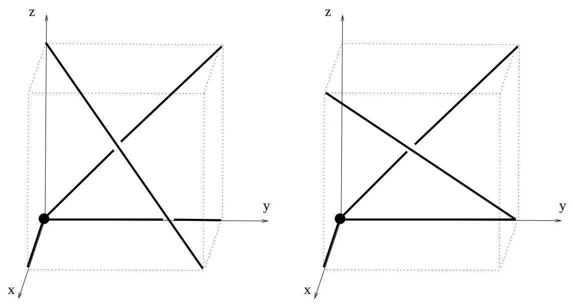

FIGURE 1. Some motifs of type $aafd$.

The resulting 2 model nets, $\mathcal{M}_1$ and $\mathcal{M}_2$ are equivalent by a rotation about the line through the centre of the cube in the x-axis direction. Thus $\mathcal{M}_1$ and $\mathcal{M}_2$ are equivalent to the model net $\mathcal{M}^g_{aad}$ for the word $a_x a_y g_x d_1$.

(iii) Assume that exactly 1 of the 4 edges of F_e is an axial edge, which we may assume lies in the x- axis. If the 3 remaining edges are the f-edges that are incident to the origin, then the transformation of $\mathcal{M}$ by the map $(x, y, z) \to (x - z, y, z)$ has type $aaad$ and so is equivalent to $\mathcal{M}^d_{\rm pcu}$. If the 3 remaining f edges are not of this form then they are either coplanar (and, as before, $\mathcal{M}$ is equivalent to $\mathcal{M}^f_{\rm pcu}$) or only 1 of these 3 edges is incident to the origin, as in Figure 2. In these cases $\mathcal{M}$ is equivalent to a model net with 2 axial edges and so the previous arguments suffice.

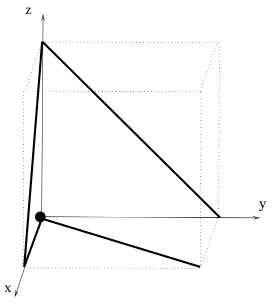

FIGURE 2. A motif with 3 non coplanar facial edges.

Thus we may assume that the defining word for $\mathcal{M}$ is of type $affd, afgd$ or $aggd$. Moreover by rotational and translational equivalence we may assume that the possible types are $a_x ffd_1, a_x fgd_1$ or $a_x ggd_1$. If all four edges are incident to the origin then $\mathcal{M}$ is equivalent by an elementary affine transformation to a model net with 2 axial edges and so there are no new cases to consider. Also if 3 edges are incident to the origin then once again the net is equivalent to the net for **hex**, and so it remains to consider the cases $a_x g_x g_y d_1, a_x g_x g_z d_1$ and $a_x g_y g_z d_1$ indicated in Figure 3.

Note that the first and third nets are the nets $\mathcal{M}^{gg}_{\rm ad}$ and $\mathcal{M}^{g_y g_z}_{\rm ad}$ in the list of model nets. That these nets are not isomorphic follows from their topological density counts. The second net has a rotation about the diagonal which is a mirror image of the first net and so is equivalent to it by elementary transformations.

(iv) Finally, for the case $m = 4$, we assume that there are no axial edges. By rotational symmetry there are 4 cases which, under the convention are uniquely specified by the words $fffd, ffgd, fggd$ and $gggd$. The last of these corresponds to a disconnected net, as we have seen in the previous section, the first gives an alternative model net for **ilc** (as we have remarked prior to the proof), and the other 2 nets, for $ffgd$ and $fggd$, are easily seen to be affinely equivalent to a model net with 1 axial edge.

The case $m = 5$. It is straightforward to see that if $\mathcal{M}$ has 3 axial edges and 2 face edges then it is equivalent to the model net $\mathcal{M}^{ff}_{\rm pcu}$ for **bct**. Also, type $aaafd$ is equivalent

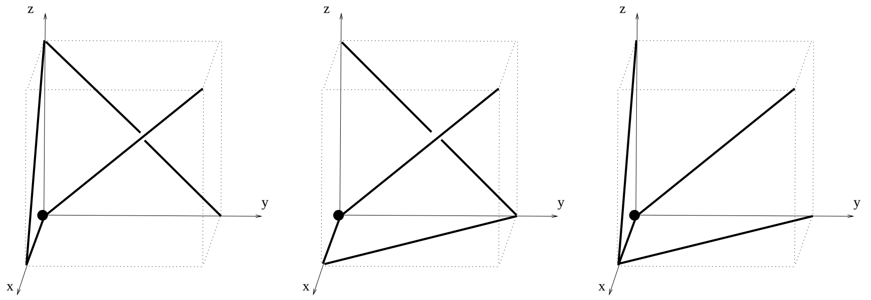

FIGURE 3. Motifs for the model nets $\mathcal{M}(a_xg_xg_yd_1), \mathcal{M}(a_xg_xg_zd_1)$ and $\mathcal{M}(a_xg_yg_zd_1)$.

to this type. On the other hand, type *aaagd* has hxl-multiplicity equal to 1, rather than 2, and so is in a new equivalence class, also with no edge penetrations. In fact this model net has topology **ile**.

Consider next the model nets with 2 axial edges and no diagonal edges. These also have no penetrating edges and are of hxl-multiplicity 1 or 2. Moreover it is straightforward to show that each is equivalent by elementary affine transformations to a model net with 3 axial edges and so they equivalent to the model nets for **bct** and **ile** respectively. The same is true for the 9 nets of type *aawd* where w is a word in 2 facial edges which is not of type gg.

Thus, in the case of 2 axial edges it remains to consider the types $a_xa_ywd_1$ with $w = g_xg_y, g_xg_z$ and g_yg_z each of which has a penetrating edge of type 4^2. The first two of these are model nets in the list and give new and distinct affine equivalence classes in view of their penetration type and differing hxl$(\mathcal{N})$ count. The third net, for the word $a_xa_yg_yg_zd_1$ is a mirror image of the first net and so is orientedly affinely equivalent to it.

It remains to consider the case of 1 axial edge, a_x, together with d_1 and 3 facial edges. If there are 2 edges of type f_x, f_y or f_z then there is an elementary equivalence with a model net with 2 axial edges. The same applies if there is a single such edge. For an explicit example consider $a_xf_xg_yg_zd_1$. The image of this net under the transformation $(x, y, z) \to (x, y - z, z)$ gives a depth 1 net with 2 axial edges. The transformation of motifs is indicated in Figure 4.

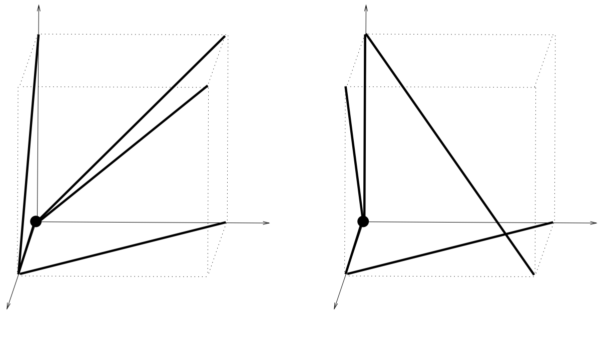

FIGURE 4. Change of motif under $(x, y, z) \to (x, y - z, z)$.

Finally the model net for $a_x g_x g_y g_z d_1$ appears in the listing and gives a new affine class with penetration type 3^2.

The case $m = 6$**.** We first assume that there is no diagonal edge in the motif for $\mathcal{M}$ and therefore no edge penetration of type 4^2 or 3^2. There are 2 distinguished model nets in the list for this case, one with the 3 facial edges of type f (a net with topology **ild**) and one where the 3 facial edges are of type g (a net with topology **fcu**). Two other choices of facial edges are possible (up to rotation) and these are readily seen to be equivalent to the **ild** and **fcu** nets.

We may now assume that there exists a diagonal edge in the standardised form of the edge word defining $\mathcal{M}$. If there are 3 axial edges then there are 3 possibilities, namely types $aaaffd, aaafgd, aaaggd$. The first 2 cases are not new, since the transformation $(x, y, z) \to (x, y - z, z)$ give motifs without a diagonal edge, while the model net for $aaaggd$ appears in the list, with penetration type 4^2 and $\mathrm{hxl}(\mathcal{M}) = 2$.

We may now assume that $\mathcal{M}$ has a standardised word $a_x a_y w d_1$ where w is a word in 3 facial edges. For w of fff type there are 3 cases, namely $f_x f_y g_z$, $f_x g_y f_z$ and $g_x f_y f_z$, each of which transforms by an elementary transformation (respectively, $x \to x - z, y \to y - z$ and $x \to x - z$) to a case with 3 axial edges. For w of type fgg there are 3 cases, namely $f_x g_y g_z$, $g_x f_y g_z$ and $g_x g_y f_z$. The first and second of these are not new, since the transformations $y \to y - z$ and $x \to x - z$, respectively, lead to an equivalence with $\mathcal{M}^{ggd}_{\mathrm{pcu}}$, while the third case is the model net $\mathcal{M}^{ggf}_{aad}$.

Finally, for w of type ggg we have the model net $\mathcal{M}^{ggg}_{aad}$.

The case $m = 7$**.** There are 4 cases of standardised edge word of the form $aaawd$ with w of type fff, ffg, fgg or ggg. The model net for $a_x a_y a_z f_x f_y f_z d$ is obtained from the model net for $a_x a_y a_z f_x f_y g_z d$ by the transformation $y \to y - z$ followed by a rotation. Thus there is a maximum of 3 equivalence classes with representative model nets $\mathcal{M}^{fffd}_{\mathrm{pcu}}, \mathcal{M}^{ggfd}_{\mathrm{pcu}}, \mathcal{M}^{gggd}_{\mathrm{pcu}}$. Since these are distinguished by their edge penetration type the proof is complete. □
